# Trihelix transcription factors GTL1 and DF1 prevent aberrant root hair formation in an excess nutrient condition

**DOI:** 10.1111/nph.18255

**Published:** 2022-06-17

**Authors:** Michitaro Shibata, David S. Favero, Ryu Takebayashi, Arika Takebayashi, Ayako Kawamura, Bart Rymen, Yoichiroh Hosokawa, Keiko Sugimoto

**Affiliations:** ^1^ RIKEN Center for Sustainable Resource Science Yokohama 230‐0045 Japan; ^2^ Division of Materials Science, Graduate School of Science and Technology Nara Institute of Science and Technology Ikoma 630‐0192 Japan; ^3^ KU Leuven Plant Institute (LPI) KU Leuven Kasteelpark Arenberg 31 Leuven B‐3001 Belgium; ^4^ Department of Biological Sciences University of Tokyo Tokyo 119‐0033 Japan

**Keywords:** *Arabidopsis thaliana*, excess nutrition, gene regulatory network, root hairs, transcription factor

## Abstract

- Root hair growth is tuned in response to the environment surrounding plants. While most previous studies focused on the enhancement of root hair growth during nutrient starvation, few studies investigated the root hair response in the presence of excess nutrients.
- We report that the post‐embryonic growth of wild‐type Arabidopsis plants is strongly suppressed with increasing nutrient availability, particularly in the case of root hair growth. We further used gene expression profiling to analyze how excess nutrient availability affects root hair growth, and found that RHD6 subfamily genes, which are positive regulators of root hair growth, are downregulated in this condition.
- However, defects in GTL1 and DF1, which are negative regulators of root hair growth, cause frail and swollen root hairs to form when excess nutrients are supplied. Additionally, we observed that the *RHD6* subfamily genes are mis‐expressed in *gtl1‐1 df1‐1*. Furthermore, overexpression of *RSL4*, an *RHD6* subfamily gene, induces swollen root hairs in the face of a nutrient overload, while mutation of *RSL4* in *gtl1‐1 df1‐1* restore root hair swelling phenotype.
- In conclusion, our data suggest that GTL1 and DF1 prevent unnecessary root hair formation by repressing *RSL4* under excess nutrient conditions.

## Introduction

Plant biomass accumulation is generally limited by the availability of nutrients, particularly that of the three primary nutrients, nitrogen (N), phosphorus (P) and potassium (K). In addition to N, P and K; the elements carbon (C), hydrogen (H), oxygen (O), calcium (Ca), magnesium (Mg) and sulfur (S) are collectively called essential macronutrients. Boron (B), chlorine (Cl), iron (Fe), manganese (Mn), molybdenum (Mo), nickel (Ni) and zinc (Zn), however, are referred to as essential micronutrients. Generally, plants cannot survive or complete their life cycle without these essential nutrients. Furthermore, aluminum (Al), silicon (Si), sodium (Na) and cobalt (Co), the so‐called beneficial elements, are known to improve growth and yield of some plant species. Fertilizer application is an effective means of increasing plant growth in conditions where nutrients are otherwise lacking (Barker & Pilbeam, 2015). However, excess fertilizer application can harm the environment in multiple ways, such as by reducing air, water and land quality as has been documented for P and N fertilizers used in parts of Europe, North America and Asia (Liu *et al*., 2010; Foley *et al*., 2011; MacDonald *et al*., 2011; Sutton *et al*., 2021). Although over‐fertilization can reduce land quality, thus making it less conducive to plant growth, our knowledge regarding plant responses to excess nutrients pales in comparison to our understanding of responses associated with nutrient starvation.

Root hairs, which grow from the epidermis, impact nutrient uptake from the soil, as they increase the surface area of the root system. In Arabidopsis, root hairs generally initiate from trichoblasts, one of two types of epidermal cells specified during root development. Atrichoblasts, however, are root epidermal cells that do not produce hairs under normal conditions (Salazar‐Henao *et al*., 2016). Root hair initiation, as well as growth of root hairs, is precisely regulated based on nutrient availability. For instance, inorganic phosphate (Pi) starvation promotes both root hair formation and growth. Under Pi starvation, root hair number is increased by the initiation of root hairs from atrichoblasts and also by the production of multiple hairs from a single epidermal cell (Ma *et al*., 2001). At the same time, root hair length increases nearly three‐fold in low compared to high Pi conditions (Bates & Lynch, 1996). This response of increased root hair growth under low P availability in Arabidopsis is important for increasing P acquisition under P‐limiting conditions (Bates & Lynch, 2000, 2001). Iron deficiency also strongly affects root hair development (Schmidt *et al*., 2000). In contrast to Pi starvation, however, Fe deficiency increases root hair branching rather than promoting ectopic root hair formation (Müller & Schmidt, 2004). These findings suggest that at least some environmental signals affect root hair development independently of each other (Schmidt & Schikora, 2001).

Several transcription factors (TFs) have been identified that play key roles regulating root hair development (Ishida *et al*., 2008; Bruex *et al*., 2012; Shibata & Sugimoto, 2019; Vissenberg *et al*., 2020). Atrichoblasts are characterized specifically by the expression of *GLABRA2* (*GL2*), which encodes a TF that functions as a negative regulator of root hair formation and is often used as a marker for nonhair cells (Di Cristina *et al*., 1996; Masucci *et al*., 1996; Lin *et al*., 2015). Conversely, the basic helix‐loop‐helix (bHLH) TFs ROOT HAIR DEFECTIVE6 (RHD6) and RHD6‐LIKE1 (RSL1) are key factors that promote hair development (Masucci & Schiefelbein, 1994; Menand *et al*., 2007; Pires *et al*., 2013). Following specification of root epidermal cells as trichoblasts, RHD6 and RSL1 promote transcription of genes encoding other bHLHs, including *RSL2*, *RSL4* and *Lotus japonicus ROOTHAIRLESS‐LIKE 3* (*LRL3*) (Masucci & Schiefelbein, 1994; Karas *et al*., 2009; Yi *et al*., 2010). The *rsl2 rsl4* double mutant completely lacks root hairs (Yi *et al*., 2010), indicating that *RSL2* and/or *RSL4* are essential for root hair growth. Similarly, the double or triple mutants for *LRL3* and its homologs *LRL1* and/or *LRL2* have short root hairs that occur at a lower density than normal (Karas *et al*., 2009; Tam *et al*., 2015; Breuninger *et al*., 2016). Thus LRL3, together with LRL1 and LRL2, contributes to both root hair formation and growth. In addition to promoting growth of root hairs in response to developmental signals, bHLHs, particularly RSL2 and RSL4, are also important for root hair growth induced by exogenous phytohormone (auxin, cytokinin, ethylene or jasmonic acid) treatments or nutrient (Pi, N or Fe) deficiency (Yi *et al*., 2010; Datta *et al*., 2015; Zhang *et al*., 2016; Feng *et al*., 2017; Mangano *et al*., 2017; Bhosale *et al*., 2018; X. Han *et al*., 2020; Qiu *et al*., 2021). Therefore, RSL2 and RSL4 appear to be core factors in the gene regulatory network (GRN) that controls root hair growth (Lee & Cho, 2013; Franciosini *et al*., 2017; Shibata & Sugimoto, 2019). In addition to these positive regulators of root hair growth, negative regulators have also been identified. The trihelix TF GT2‐LIKE1 (GTL1) and its closest homolog, DF1, terminate root hair growth by directly repressing *RSL4* together with RSL4 target‐genes (Shibata *et al*., 2018). In addition, a DOF‐type TF, OBF BINDING PROTEIN 4 (OBP4), is a negative regulator of root hair growth, as induction of *OBP4* reduces root hair length (Rymen *et al*., 2017). Unlike GTL1 and DF1, OBP4 represses *RSL2* expression and does not affect *RSL4* expression, suggesting that plants have at least two transcriptional pathways that repress root hair growth.

Here we investigated how Arabidopsis root hairs are affected by the presence of multiple nutrients in excess. Specifically, we demonstrate that root hair growth is strongly suppressed on double‐strength Murashige–Skoog (2×MS) medium. Further, we show that the *gtl1‐1 df1‐1* mutant forms frail root hairs on 2×MS, suggesting that GTL1 and DF1 prevent aberrant root hair development in the presence of excess nutrients. These findings shed light on the mechanisms that plants have evolved to adapt to growth in variable conditions.

## Materials and Methods

### Plant materials and growth conditions

The *gtl1‐1*, *df1‐1*, *rsl4‐1*, *rhd6‐3*, *obp4‐2*, *obp4‐3*, *pGTL1:GTL1‐GFP*, *pEXP7:GTL1‐GFP*, *pGL2:GL2‐GFP/gl2‐8*, and *pEXP7:NLS‐GFP* lines were previously described (Breuer *et al*., 2009, 2012; Yi *et al*., 2010; Ikeuchi *et al*., 2015; Rymen *et al*., 2017; Shibata *et al*., 2018). *lrl3‐2* corresponds to SALK_012380 (Supporting Information Fig. S13). *35S:XVE>>RHD6* (Coego *et al*., 2014) was obtained from ABRC (Columbus, OH, USA). Both *RSL4* complementary DNA (cDNA) and genomic DNA (gDNA) overexpression lines were generated for this study. See Methods S1 and Table S1 for the detail.

Plants were grown at 22°C, with 60% relative humidity and under continuous light (50–70 μmol m^−2^ s^−1^). Half‐strength MS (1/2×MS) or 2×MS media used for plant growth were prepared following the recipe shown in Table S2. For self‐made MS media (Table S3), each chemical solution was prepared and mixed before autoclaving. Iron sulfate (FeSO_4_) was added together with ethylenediaminetetraacetic acid disodium salt (2Na‐EDTA) to prevent precipitation.

### Plant growth analysis

For the measurements of primary root length and lateral root length, root images from 14 d‐old seedlings were taken using a scanner (GT‐X830; Seiko Epson, Suwa, Japan). Subsequently, the shoot tissues from the same seedlings were collected for fresh weight measurements. Material harvested from five seedlings was pooled and weighed on a microbalance (AT200; Mettler Toled, Columbus, OH, USA). To measure root hair length, images were taken from primary roots of 7 d‐old seedlings with a dissection microscope (M165 FC equipped with a DFC 7000T; Leica Microsystems, Wetzlar, Germany). The region within *c*. 7 mm from the root tip was used for quantification. The mean value from the length of the 20 longest root hairs from each seedling was adopted as the ‘root hair length’ for that seedling. The ‘root hair length’ was determined using this approach in at least 12 seedlings for each condition. All experiments were done at least three times to confirm reproducibility. All image analysis was manually performed using ImageJ (v.1.53g).

### Microscopic observation

Roots from 1 wk‐old Arabidopsis plants were stained with 50 μg ml^−1^ (w/v) propidium iodide (PI; Invitrogen). Expression patterns of *pEXP7:NLS‐GFP* and *pGL2:GL2‐GFP* were then obtained using a Leica SP5 confocal laser scanning microscope equipped with an HC PL APO 20× 0.7 dry objective and the Leica HyD hybrid detector. Light of wavelength 488 nm from an argon‐ion laser was used for excitation and emitted light of wavelengths from 593 to 655 nm for PI and 500 to 568 nm for green fluorescent protein (GFP), respectively, were detected using a spectral detector. Image quantification is described in Methods S1.

### Atomic force microscopy (AFM)

Root sections from 1‐wk‐old seedlings were used for measuring root hair strength. The samples were immobilized by molten 1.5% low‐melting point agarose (Agarose LMT 1‐20K, PrimeGel; Takara Bio, Kusatsu, Japan) spread thinly on a glass‐bottom dish and immediately covered in water. Intact root hairs were selected for measurement with an inverted microscope (IX71; Olympus, Tokyo, Japan). To reduce the effect of leverage, a distance of 30 μm from the root hair base was used as the contact point. Data were obtained using an atomic force microscopy (AFM) system (Nanowizard 4; JPK Instruments, Berlin, Germany), to which an AFM cantilever (SD‐Sphere‐NCH, force constant: 42 N m^−1^; Nanosensors, Neuchatel, Switzerland) was attached. The cantilever tip is shaped into a hemisphere (400 nm radius) to avoid damaging the contact point on the cell. Force curves were observed using contact mode with set point at 130 nN and approach velocity at 2.0 μm s^−1^. The data were analyzed by the Hertz model, using JPK Data Processing (v.6.1.158) software, in which the apparent elastic modulus of the cell (stiffness score) was estimated by Young's modulus.

### RNA extraction and RT‐qPCR analysis

Total RNA was extracted from 7‐d‐old roots using the RNeasy Plant Mini Kit (Qiagen). Extracted RNA was reverse transcribed using a PrimeScript RT reagent kit with gDNA Eraser (Takara Bio) in accordance with the accompanying protocol. Transcript levels were determined via quantitative polymerase chain reaction (qPCR) using the Thunderbird SYBR qPCR Mix kit (Toyobo, Osaka, Japan) and Mx399P QPCR system (Agilent, Santa Clara, CA, USA). The expression of either the *UBQ10* or *HEL* gene was used as an internal control. The set of primers is provided in Table S4.

### Promoter‐luciferase assay

The constructs shown in Fig. S10(a) were introduced into Arabidopsis MM2d culture cells (Menges & Murray, 2002) by a gold particle bombardment system (Bio‐Rad). Luciferase activity was quantified using a Mithras LB940 microplate luminometer (Berthold Technologies, Bad Wildbad, Germany) as described previously (Hiratsu *et al*., 2002). For details of plasmid construction, see Methods S1 and Table S5.

### Co‐immunoprecipitation (Co‐IP)

Full‐length cDNA of *EGFP*, *GTL1* or *RHD6* was fused with *3xFLAG*, *3xFLAG* or *3xHA* by PCR, respectively, and introduced into pEAQ‐HT‐DEST1 (Sainsbury *et al*., 2009). The resulting plasmids, *EGFP‐3xFLAG* or *GTL‐3xFLAG*, were co‐injected with *RHD6‐3xHA* into tobacco leaves by agroinfiltration. The following co‐immunoprecipitation (Co‐IP) assay was performed as described previously with some modifications (Kadota *et al*., 2016). The detailed procedure is described in Methods S1.

## Results

### Excess nutrients inhibit Arabidopsis growth

MS medium (Murashige & Skoog, 1962) is widely used for plant growth. For Arabidopsis, 1/2×MS or full‐strength MS (1×MS) media are commonly used as ‘normal’ growth conditions. Thus, in addition to 1/2×MS, we prepared 2×MS medium as an ‘excess nutrient’ condition to study the impact of excess nutrients on post‐embryonic development of Arabidopsis.

**Fig. 1:**
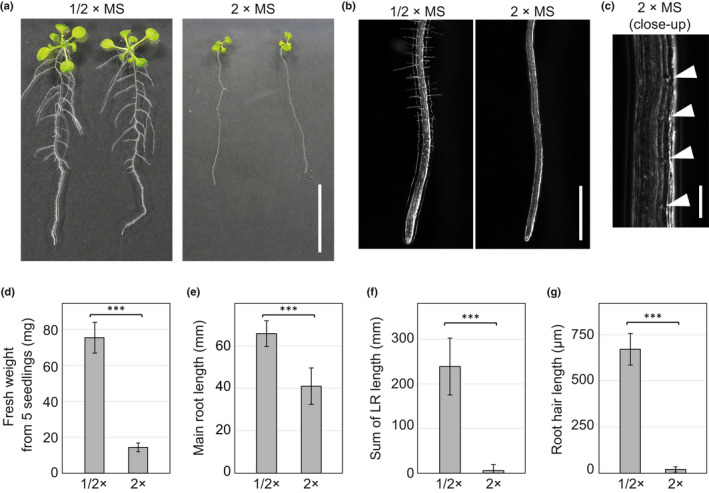
Arabidopsis growth is repressed by a higher concentration of Murashige–Skoog (MS) medium. (a) Images of 2‐wk‐old wild‐type (WT) plants grown on half‐strength MS (1/2×MS) or double‐strength MS (2×MS) medium. Bar, 1 cm. (b) Images of root tips of 1‐wk‐old WT Arabidopsis on 1/2×MS or 2×MS medium. A high‐magnification image of a root grown on 2×MS medium is shown in (c). The arrow heads in (c) show root hair initiation sites from epidermal cells. Bars: (b) 1000 μm; (c) 100 μm. Quantitative analysis of fresh weight from five seedlings (d), primary root length (e), sum of lateral root (LR) length (f) and the average length of 20 longest root hairs from each seedling (g). Data are mean ± SD (*n* = 8 for (d), 16 for (e) and (f), 12 for (g)). Asterisks indicate a significant difference (Student's *t*‐test, ***, *P* < 0.001).

As shown in Fig. 1, plant growth was severely affected by MS strength. Consistent with the visible effect on growth, the fresh (shoot) weight of 2‐wk‐old seedlings significantly decreased with higher MS strength (Fig. 1d). Although the primary root length was significantly altered between the 1/2×MS and 2×MS conditions, the total lateral root length more obviously declined with higher MS strength (Fig. 1e,f). This observation is consistent with previous reports describing that plants generally reduce lateral root development in the presence of excess nutrients in order to decrease nutrient uptake (Gruber *et al*., 2013).

### Double‐strength MS medium suppresses root hair growth

**Fig. 2:**
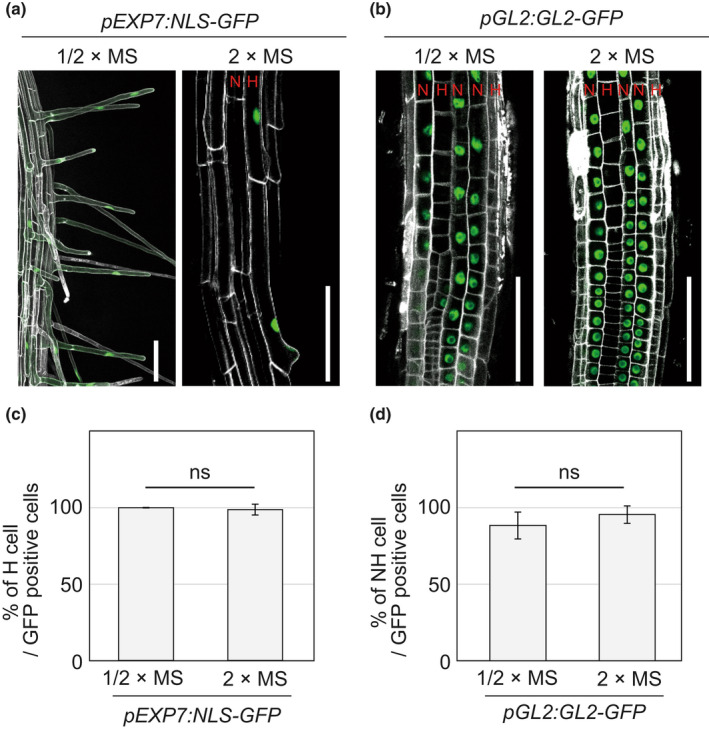
Arabidopsis root hair and nonhair cell fates are unchanged on double‐strength Murashige–Skoog (2×MS) medium. (a, b) Confocal microscope images of *pEXP7:NLS‐GFP* (a) and *pGL2:GL2GFP/gl2‐8* (*pGL2:GL2‐GFP*) (b) roots on half‐strength MS (1/2×MS) or 2×MS. *EXP7* and *GL2* are used as hair cell and nonhair cell markers, respectively. Bar, 100 μm. (c, d) The bar graph shows ratio of hair (H) cells (c) and nonhair (NH) cells (d) per green fluorescent protein (GFP)‐positive cells, respectively. The data consist of 109 cells from six seedlings in *pEXP7:NLS‐GFP* on 1/2×MS, 81 cells from eight seedlings in *pEXP7:NLS‐GFP* on 2×MS, 280 cells from six seedlings in *pGL2:GL2‐GFP* and 217 cells from six seedlings in *pGL2:GL2‐GFP*. ns above the graph indicates a not significant result from Student's *t*‐test. Error bars indicate SD.

In addition to affecting lateral roots, MS concentration also affected root hairs (Fig. 1b,c,g). To study the impact of excess nutrition on root hair development in greater detail, we observed root hairs under high magnification (Fig. 1b). Quantitation of root hair length revealed a substantial decrease in root hair growth on 2×MS medium (Fig. 1g). Two weeks after germination, only extremely short root hairs were observed on 2×MS medium. Close inspection of root hairs on 2×MS medium revealed dome‐like structures on epidermal cells, indicating that root hair initiation had progressed (Fig. 1c). To confirm that root hair/nonhair cell identities remain unchanged on 2×MS, we observed the expression patterns of *EXPANSIN A7* (*EXPA7*) and *GL2*, which are hair cell and nonhair cell markers, respectively (Masucci *et al*., 1996; Cho & Cosgrove, 2002). In the case of Arabidopsis, since hair cells and nonhair cells are tandemly arranged within cell files in the root epidermis, GFP signals from both *pEXP7:NLS‐GFP* and *pGL2:GL2‐GFP* were observed in strips (Fig. 2a,b). Also, hair cells and nonhair cells are distinguishable regardless of bulge formation based on cell length (Dolan *et al*., 1994). Since nonhair cells expand earlier than hair cells, cells shorter than their adjacent cells are identified as hair cells (Berger *et al*., 1998). On both 1/2×MS and 2×MS media, each GFP signal aligned within cell files of its corresponding cell type, indicating that the root hair and nonhair cell pattern is not altered by MS concentration (Fig. 2). In addition, we also examined the expression levels of *EXPA7* and *GL2* (Fig. S1). For *EXPA7*, its expression decreased on 2×MS. This is reasonable because *EXPA7* contributes to cell expansion (Cho & Cosgrove, 2002) and does not necessarily imply that there are fewer hair cells in this condition. Importantly, *GL2* expression was also reduced on 2×MS (Fig. S1). This suggests that the extreme short root hair response is not caused by an increase of nonhair cells in the root epidermis due to an increase of the GL2 root hair identity repressor. Taken together, these results indicate that the root hair phenotype on 2×MS media is caused by defects in cell growth rather than cell specification.

### A combination of multiple nutrients alters root hair growth

**Fig. 3:**
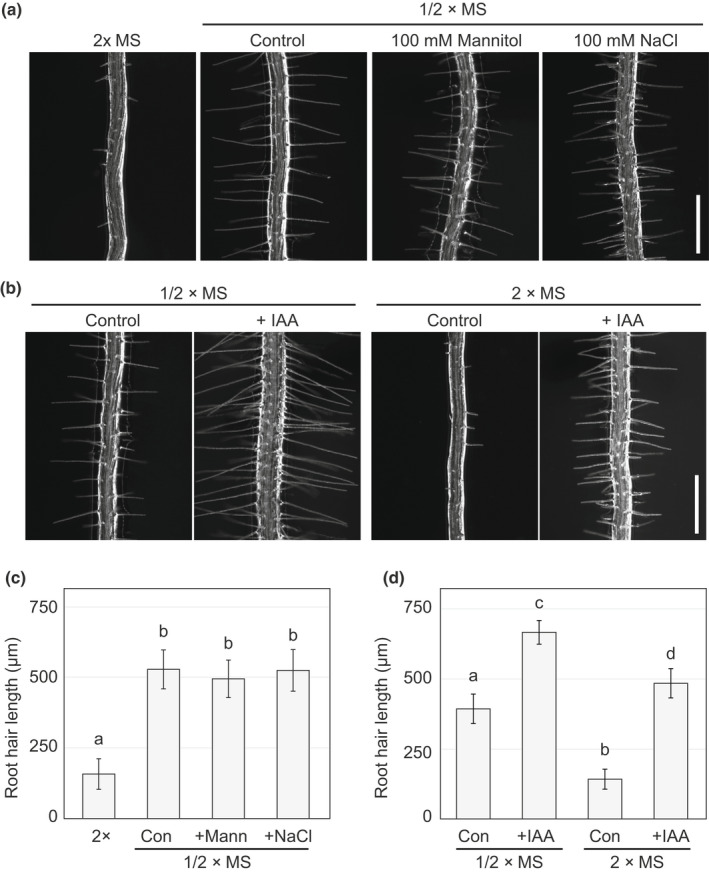
Wild‐type (WT) Arabidopsis plants purposefully suppress root hair growth on double‐strength Murashige–Skoog (2×MS) medium. (a, b) Microscopic images of root hairs. WT seedlings were grown on half‐strength MS (1/2×MS) for 5 d and then transferred to the indicated conditions. The images were recorded 2 d after transfer. 100 mM Mannitol (+Mann) and 100 mM sodium chloride (+NaCl) were added to 1/2×MS to produce osmotic stress and salt stress conditions, respectively in (a). Dimethyl sulfoxide (DMSO) (Con) or 1 nM auxin (indole‐3‐acetic acid (+IAA)) were added to 1/2×MS or 2×MS in (b). Note that WT seedlings produced normal root hairs in response to IAA treatment even on 2×MS. Bar, 500 μm. (c, d) Quantitative data for the average length of 20 longest root hairs from 12 seedlings. Data are mean ± SD. Different letters indicate significant differences between media conditions (one‐way ANOVA with *post hoc* Tukey HSD test, *P* < 0.05).

To further study the root hair phenotype on 2×MS, wild‐type (WT) plants were grown on 1/2×MS media with 100 mM Mannitol or 100 mM sodium chloride (NaCl), which confers much stronger osmotic or salt stress, respectively, than 2×MS. To minimize the possibility of macroscopic growth effects indirectly affecting root hairs, 5 d‐old seedlings grown on 1/2×MS were transferred to the experimental media and incubated for an additional 2 d prior to observation. Initially, we confirmed that seedlings transferred to 2×MS showed similar root hair phenotypes, although the growth inhibition of root hairs was slightly mitigated (Fig. 3a). However, the osmotic and salt stress conditions did not affect root hair length compared to normal 1/2×MS media (Fig. 3a,c). These results indicate that the root hair phenotype on 2×MS is not caused by these types of external stress. To study whether the WT can form normal root hairs on 2×MS, we grew WT on 2×MS containing a natural auxin (indole‐3‐acetic acid (IAA)), which promotes root hair growth (Fig. 3b,d). The hair length of seedlings grown on 2×MS was shorter than those grown on 1/2×MS; however, the WT formed normal root hairs in response to exogenous auxin. These series of experiments strongly suggest that WT plants are physically able to form root hairs on 2×MS but purposefully suppress their growth in this condition.

To identify which nutrient(s) cause the strong suppression of root hair growth on 2×MS medium, we prepared custom MS media and altered the ingredients. Initially, we prepared a medium named ‘1/2×MS_all’, which mimics 1/2×MS made from an MS salt mix (Table S1), and confirmed that root hair growth was similar to normal 1/2×MS (Fig. S2). Next, to identify which nutrients cause the strong suppression of root hair growth on 2×MS, we prepared various MS media, each in which one ingredient is increased from the 1/2×MS level to that of 2×MS (Table S2). Among 14 different chemicals found in MS salts, we found that increasing Mg (1/2×MS_2×MgSO_4_), P (1/2×MS_2×KH_2_PO_4_), Fe (1/2×MS_2×FeSO_4_ + 2×EDTA) or Mn (1/2×MS_2×MnSO_4_) sources significantly reduced root hair length compared to 1/2×MS_all. However, none of these single nutrient increases suppressed root hair growth as severely as 2×MS (Fig. S2).

Next, we prepared a medium named ‘2×MS_all’, which mimics 2×MS made from an MS salt mix (Table S1), and various 2×MS media, each in which one ingredient is reduced from the 2×MS level to that of 1/2×MS (Table S2). Initially, we confirmed that root hair growth was strongly inhibited in WT plants grown on 2×MS_all (Fig. S3). Next, using various 2×MS media, we examined which nutrients, when reduced to the level found in 1/2×MS, mitigate the strong suppression of root hair growth. Among 14 different chemicals found in MS salts, we found that the reduction of nitrate (2×MS_1/2×KNO_3_) or iodine (2×MS_1/2×KI) sources partially restored root hair growth (Fig. S3). Further, to evaluate the impact of multiple N sources, including one containing ammonium, on root hair growth, we prepared medium with reduced potassium nitrate (KNO_3_) and ammonium nitrate (NH_4_NO_3_). Although reduction of only NH_4_NO_3_ (2×MS_1/2×NH_4_NO_3_) did not increase root hair length, decreased levels of both KNO_3_ and NH_4_NO_3_ (2×MS_1/2×KNO_3_ + 1/2×NH_4_NO_3_) further restored root hair growth compared to 2×MS_1/2×NH_4_NO_3_ (Fig. S3). Therefore, to investigate if double‐strength N sources (KNO_3_ and NH_4_NO_3_) are sufficient to suppress root hair formation, we observed root hair phenotypes in plants grown on 1/2×MS media with 2×MS levels of both KNO_3_ and NH_4_NO_3_ (1/2×MS_2×KNO_3_ 2×NH_4_NO_3_). As opposed to reduction of these nutrients in 2×MS, addition of these nutrients to 1/2×MS did not clearly affect root hair growth (Fig. S2). In addition to these effects resulting from decreased N sources in 2×MS, we found that reduction of Pi (2×MS_1/2×KH_2_PO_4_) affected root hair morphology. On 2×MS_1/2×KH_2_PO_4_ medium, the root hair length was comparable to the control condition, however some root hairs were swollen like a balloon (Fig. S3). Swollen root hairs were also observed on 2×MS_1/2×MnSO_4_, although the phenotype was milder.

In summary, our series of experiments using various types of MS media demonstrated that oversupply of a single nutrient does not explain the root hair phenotype observed on 2×MS_all. Therefore, we conclude that WT Arabidopsis suppress root hair growth in response to a combination of multiple nutrients, including N, P, Mg, Fe, Mn and iodine.

### GTL1 and DF1 contribute to the proper termination of root hair growth on 2xMS

**Fig. 4:**
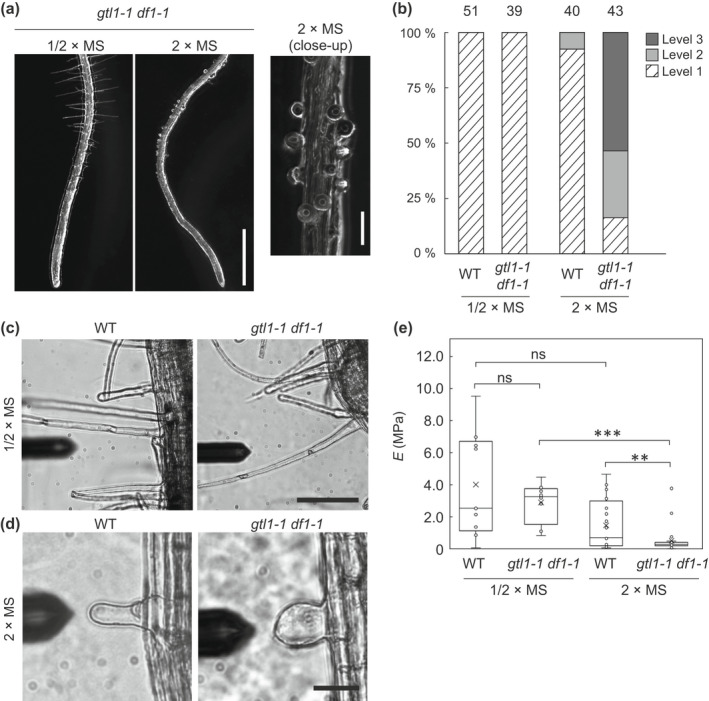
GTL1/DF1 maintain root hair stiffness on double‐strength Murashige–Skoog (2×MS) medium in *Arabidopsis thaliana*. (a) Images of root tips of 1 wk‐old *gtl1‐1 df1‐1* on half‐strength MS (1/2×MS) and 2×MS medium. A high‐magnification image of a root grown on 2×MS medium is shown on the right. Bars: (left panel) 1000 μm; (right panel) 100 μm. (b) Quantitative data for root hair morphology in wild‐type (WT) or *gtl1‐1 df1‐1* seedlings grown on the two different types of media. The number above each bar graph indicates the numbers of seedlings used for the quantification. For definition of levels, refer to Supporting Information Fig. S5. (c, d) Microscopic images of root hairs used for atomic force microscopy (AFM) analysis. The cantilever indicates the root hairs used for measurement. Bars: (c) 100 μm; (d) 30 μm. (e) Box plots showing root hair stiffness scores, as determined from AFM analysis. The boxes show the lower quartile and upper quartile values. The whiskers show the maximum and minimum values that are still within a factor of 1.5 times the interquartile range beyond each quartile. The horizontal bar and the cross in each box indicate the median and mean value, respectively. The *y*‐axis indicates Young's modulus (*E*). Asterisks indicate a significant difference (Student's *t*‐test, **, *P* < 0.01; ***, *P* < 0.001; ns, not significant).

Regarding negative regulators of root hair growth, a DOF‐type TF, OBP4, and the trihelix‐type TFs GTL1 and DF1 have been identified (Rymen *et al*., 2017; Shibata *et al*., 2018). Therefore, we hypothesized that OBP4, GTL1 and DF1 might contribute to the root hair phenotype on 2×MS medium. To test this hypothesis, we analyzed root hair growth in *obp4* mutants and the *gtl1‐1 df1‐1* mutant grown on 1/2×MS and 2×MS media. As shown in Fig. 4(a), the *gtl1‐1 df1‐1* mutant forms root hairs with normal morphology on 1/2×MS. However, in the presence of 2×MS, we found that *gtl1‐1 df1‐1* formed tiny root hairs (Figs 1b, 4a). Furthermore, most root hairs found in *gtl1‐1 df1‐1* grown on 2×MS were swollen and a few hairs had ruptured (Figs 4a,b, S4). These results suggest that GTL1 and DF1 are required for proper termination of root hair growth and/or maintenance of the tube‐like structure of root hairs on 2×MS medium.

In contrast, analysis of *obp4* mutants indicates that this gene does not contribute to the root hair phenotype on 2×MS. We examined *obp4‐2* (knockdown allele) and *obp4‐3* (knockout allele) mutants and found that both showed root hair responses similar to that of the WT (Fig. S5). Both *obp4‐2* and *obp4‐3* mutants suppressed root hair growth on 2×MS and did not form swollen root hairs, in contrast to *gtl1‐1 df1‐1* (Figs 4a,b, S5). Thus, we used the *gtl1‐1 df1‐1* mutant to further study the molecular mechanisms involved in responses to 2×MS.

### Swollen root hairs in *gtl1‐1 df1‐1* lose stiffness

To further investigate the morphological differences between WT and *gtl1‐1 df1‐1* root hairs, we estimated the stiffness scores of root hairs from analysis of the force‐indentation curves acquired by AFM measurement. Initially, we confirmed root hair stiffness in plants grown in a normal nutrient condition, i.e. on 1/2×MS medium. In the case of the 1/2×MS condition, no significant difference in the AFM measurement was detected between the WT and *gtl1‐1 df1‐1* (Figs 4c,d, S6). The resulting score for the WT was 4.04 ± 3.31 MPa, which was higher than the score obtained from primary roots (0.47 ± 0.12 MPa) (Fig. S6b,c).

In contrast, on 2×MS, an obvious difference in root hair stiffness was observed in *gtl1‐1 df1‐1* compared to WT (Fig. 4c,d). More specifically, the majority of root hairs in WT were stiff, while a few were loose. In contrast, most *gtl1‐1 df1‐1* root hairs were loose and only a few were stiff (Fig. 4d). Compared to primary roots, root hairs in the WT were much stiffer, whereas root hairs and primary roots produced similar stiffness readings in *gtl1‐1 df1‐1* (Fig. S6b,c). These data indicate that, besides being swollen, the root hairs in *gtl1‐1 df1‐1* have lost stiffness typically associated with root hairs.

### The expression of key TFs for root hair growth are downregulated on 2xMS

Based on the root hair phenotypes, we concluded that WT plants suppress root hair growth on 2×MS via the activity of GTL1 and DF1. Thus, we tested if *GTL1* and *DF1* are induced by higher MS concentrations. However, messenger RNA (mRNA) levels of both *GTL1* and *DF1* actually declined on 2×MS compared to 1/2×MS (Fig. S7). We next examined GTL1 and DF1 protein levels using *pGTL1:GTL1‐GFP/gtl1‐1* and *pDF1:DF1‐GFP/df1‐1* lines, respectively. Since both GTL1‐GFP and DF1‐GFP were broadly observed in roots, we examined only the early stage of the root hair zone for quantification of protein levels. As shown in Fig. S8, we did not detect significant differences in accumulation of either GTL1‐GFP or DF1‐GFP between 1/2×MS and 2×MS from our imaging analysis.

**Fig. 5:**
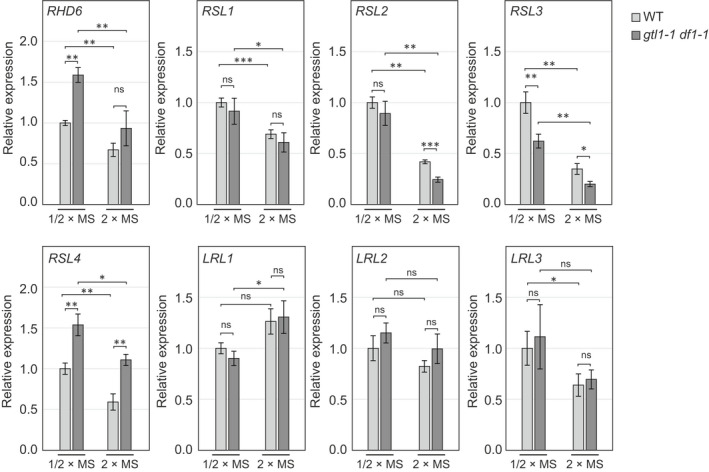
The expression of Arabidopsis root hair regulators is affected by Murashige–Skoog (MS) medium. Reverse transcription quantitative polymerase chain reaction (RT‐qPCR) analysis of key transcription factors regulating root hair growth in wild‐type (WT) and *gtl1‐1 df1‐1* grown on half‐strength MS (1/2×MS) or double‐strength MS (2×MS) media. Expression levels are normalized to that of the *HEL* gene and shown as a relative value compared to WT grown on 1/2×MS. Data are mean ± SD (*n* = 3, biological replicates). Asterisks indicate a significant difference (Student's *t*‐test, *, *P* < 0.05; **, *P* < 0.01; ***, *P* < 0.001; ns, not significant).

To study the activity of GTL1 and DF1 as TFs, we next examined the gene expression profiles of WT and *gtl1‐1 df1‐1* roots on 1/2×MS or 2×MS. Since members of the RHD6 subfamily generally play key roles promoting root hair growth in response to environmental signals, we investigated if the expression of the genes encoding these TFs is affected by MS strength. Between the WT and *gtl1‐1 df1‐1*, we found that *RHD6*, *RSL2*, *RSL3* and *RSL4* were differentially expressed in at least one of the two conditions (Fig. 5). Among these genes, *RHD6* and *RSL4* were expressed at higher levels in *gtl1‐1 df1‐1*, which is consistent with our previous data (Shibata *et al*., 2018). Interestingly, the expression of *RHD6* and *RSL4* was reduced on 2×MS compared to 1/2×MS. These expression data are consistent with the near lack of root hair growth in the WT on 2×MS. However, the expression levels of *RHD6* and *RSL4* in *gtl1‐1 df1‐1* on 2×MS were similar to those in the WT on 1/2×MS, suggesting that *RHD6* and *RSL4* in *gtl1‐1 df1‐1* are expressed at a similar level on 2×MS as in ‘normal’ conditions (Fig. 5).

In contrast to *RHD6* and *RSL4*, *RSL2* and *RSL3* levels were lower in *gtl1‐1 df1‐1* compared to the WT. Notably, the expression of both *RSL2* and *RSL3* was significantly reduced in *gtl1‐1 df1‐1* on 2×MS (Fig. 5), indicating that loss of function of GTL1 and DF1 causes stronger suppression of *RSL2* and *RSL3* expression on 2×MS. However, similar to both *RHD6* and *RSL4*, the levels of *RSL2* and *RSL3* declined on 2×MS compared to 1/2×MS in both WT and *gtl1‐1 df1‐1*.

Since the *gtl1‐1 df1‐1* mutant still exhibited reduced expression of these *RHD6* subfamily genes on 2×MS, this demonstrates that other suppression mechanisms are still active. OBP4 was characterized as a suppressor of *RSL2* and *RSL3* that inhibits root hair growth (Rymen *et al*., 2017). To study whether OBP4 is required for the repression of *RSL2* and *RSL3* on 2×MS, we examined the expression levels of *RSL2* and *RSL3* on 2×MS in both *obp4* mutants. However, the expression levels of these genes in *obp4‐2* and *obp4‐3* were similar to those observed in the WT (Fig. S9). Collectively, these results indicate that the decline of *RSL2* and *RSL3* expression on 2×MS is independent from OBP4.

### GTL1 dominantly suppresses activation of *RSL4* by RHD6

In a previous study employing chromatin immunoprecipitation with DNA microarray (ChIP‐chip), we showed that GTL1 directly represses *RHD6* and *RSL4* to terminate root hair growth (Shibata *et al*., 2018). Thus, in this study, we validated the hypothesis that *RHD6* and *RSL4* are regulated by GTL1 and DF1 using a promoter‐luciferase assay employing MM2d, an Arabidopsis cell culture line. We found that *GTL1* or *DF1* overexpression strongly reduced *RHD6* and *RSL4* promoter activities compared to the control (empty) vector (Fig. S10), demonstrating that *RHD6* and *RSL4* are under the control of both GTL1 and DF1.

**Fig. 6:**
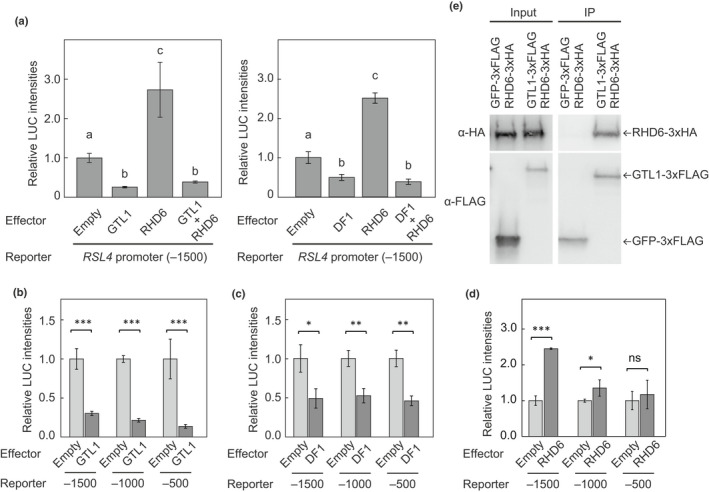
GTL1 dominantly represses *RSL4* expression in *Arabidopsis thaliana*. (a) The effect of RHD6, GTL1 or DF1 on *RSL4* promoter activity was determined in an MM2d‐based promoter‐luciferase assay. The *RSL4* promoter region (1500 bp upstream from the start codon) fused with luciferase was used as a reporter. ‘empty’ indicates the empty effector vector was used as a negative control. The right‐most bar in each graph indicates co‐bombardment assay with *RHD6* and *GTL1* or *DF1*. Data are mean ± SD (*n* = 3). Different letters indicate significant differences (one‐way ANOVA with *post hoc* Tukey HSD test, *P* < 0.01) compared to the control vector (empty). For details regarding the plasmids used for these experiments, see Supporting Information Fig. S10. (b–d) Promoter‐luciferase assay using a series of truncated *RSL4* promoter fragments. Approximately 1500, 1000 and 500 bp upstream regions from the start codon were used. *GTL1*, *DF1* and *RHD6* were used as effectors in (b), (c) and (d), respectively. Data are mean ± SD (*n* = 3). Asterisks indicate significant differences compared to vector control (Student's *t*‐test, *, *P* < 0.05; **, *P* < 0.01; ***, *P* < 0.001; ns, not significant) (e) Co‐immunoprecipitation of RHD6 and GTL1. Each construct was introduced into tobacco leaves by agroinfiltration. Protein extracts from leaves transiently expressing the indicated proteins were immunoprecipitated with anti‐FLAG magnetic beads and then probed via Western blot using an anti‐HA antibody. GFP‐3xFLAG was used as a negative control. The left panel and the right panel indicate results for input and immunoprecipitation samples, respectively. The unedited membrane images are shown in Fig. S11.

Next, since *RSL4* is known as a direct target of RHD6 and GTL1 (Yi *et al*., 2010; Shibata *et al*., 2018), we studied the relationship between GTL1, DF1 and RHD6 on *RSL4* promoter activity. As shown in Fig. 6(a), we reproduced that RHD6 and GTL1/DF1 work as an activator and a repressor of *RSL4* promoter activity, respectively, in culture cells, consistent with their effects on *RSL4* expression *in planta* (Yi *et al*., 2010; Shibata *et al*., 2018). When both types of TFs are introduced together into culture cells, we found that GTL1/DF1 dominantly represses *RSL4* promoter activity (Fig. 6a). To identify the regions within the *RSL4* promoter important for GTL1‐/DF1‐ and RHD6‐mediated regulation, a truncated series of *RSL4* promoters were used in another promoter‐luciferase assay. When we used a 1500 bp region directly upstream from the start codon of *RSL4*, both RHD6 and GTL1/DF1 altered *pRSL4*:*LUC* activity (Fig. 6b–d). However, when the fragment was shortened to contain only 500 bp immediately upstream of the *RSL4* start codon, RHD6 no longer activated *pRSL4*:*LUC*, while GTL1/DF1 still repressed expression of the reporter (Fig. 6b–d). These results suggest that RHD6 and GTL1/DF1 can regulate *RSL4* individually. Also, these data showed that GTL1 and DF1 function somewhat similarly as TFs. GTL1 is known to bind the GT3‐box (GGTAAA) (Breuer *et al*., 2012). However, there are no GT3‐boxes within the region 500 bp upstream from the *RSL4* start codon. To identify the region bound by GTL1 and DF1 on *RSL4* promoter, we cloned 350, 300, 250 and 150 bp regions from the start codon and prepared expression vectors for promoter‐luciferase assay. However, since basal expression levels declined when these short promoter fragments were used, we could not obtain reliable results using the cell culture system. Given that DF1 has been reported to bind an atypical DNA motif, DE1 (TACAGT) (Nagano *et al*., 2001), DF1 and also GTL1 might regulate downstream genes, such as *RSL4*, by binding to unidentified motifs in their promoters.

To further study the relationship between GTL1 and RHD6, we next performed a Co‐IP assay to test if the two proteins interact with each other. The coding region of *RHD6* fused with *3xHA* and *GTL1* fused with *3xFLAG* were transiently expressed in tobacco leaves using the *Agrobacterium* infiltration method. Tobacco co‐expressing *GFP* fused with *3xFLAG* and *RHD6‐3xHA* was used as a negative control. We found when *RHD6‐3xHA* and *GTL1‐3xFLAG* were co‐expressed in tobacco leaves, RHD6 and GTL1 proteins were both detected in the immunoprecipitated sample (Figs 6e, S11). However, RHD6 protein was not detected in the immunoprecipitated sample where *RHD6‐3xHA* and *GFP‐3xFLAG* were co‐expressed (Figs 6e, S11). These data indicate that GTL1 and RHD6 form a protein complex, and also suggest that GTL1 regulates *RSL4* expression by a physical interaction with RHD6. Given the promoter‐luciferase data, GTL1 (and presumably DF1) is suggested to regulate *RSL4* in both RHD6‐independent and ‐dependent manners.

### Overexpression of *RSL4* induces abnormal root hairs on 2xMS

Given that *RHD6* and *RSL4* promote root hair growth and are repressed by GTL1/DF1 *in planta*, mis‐expression of these genes in *gtl1‐1 df1‐1* may cause the root hair swelling observed in this mutant on 2×MS. To test this hypothesis, we generated *rhd6‐3 gtl1‐1 df1‐1* and *rsl4‐1 gtl1‐1 df1‐1* triple mutants. Since we observed that *LRL3* expression declines in the WT on 2×MS compared to 1/2×MS, *lrl3‐2 gtl1‐1 df1‐1* was also generated. Then, root hair phenotypes were examined on 2×MS.

**Fig. 7:**
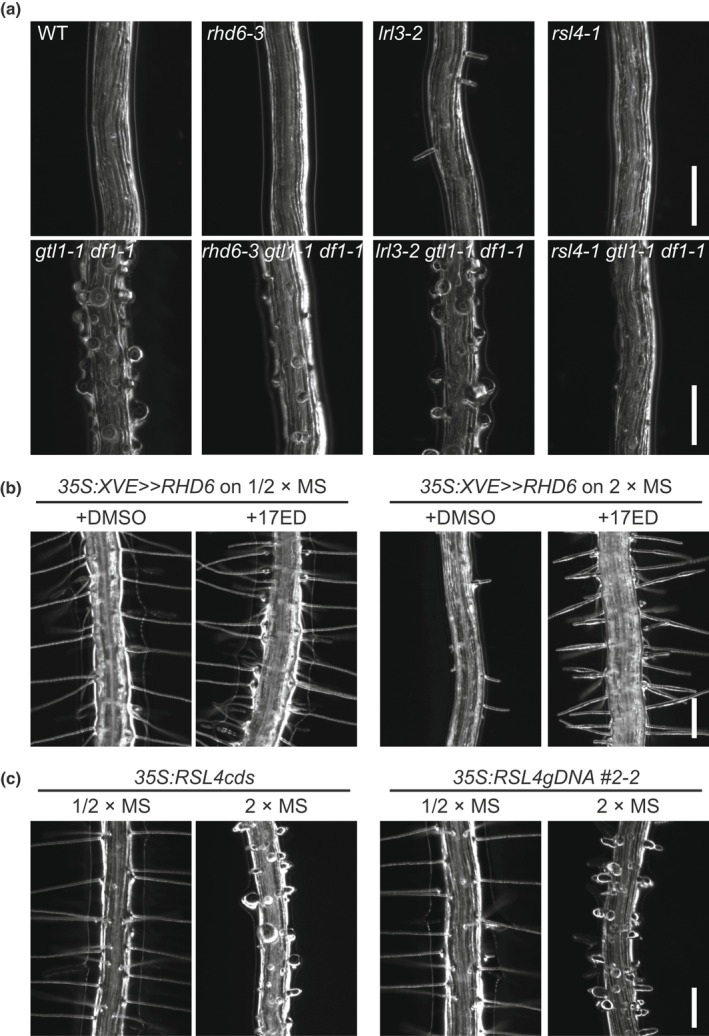
RSL4 causes aberrant root hair formation on double‐strength Murashige–Skoog (2×MS) medium in *Arabidopsis thaliana*. (a) Root hairs of *rhd6‐3*, *lrl3‐2* and *rsl4‐1* mutants in the wild‐type (WT) (upper panel) or *gtl1‐1 df1‐1* (lower panel) background grown on 2×MS medium. Note that only the *rsl4* mutation mitigated the root hair swelling phenotype of *gtl1‐1 df1‐1*. (b) Formation of normal root hairs on both 1/2×MS and 2×MS in plants after induced overexpression of *RHD6*. Dimethyl sulfoxide (DMSO) was used as a control treatment. These images were uniquely taken from bottom side of the medium. For *RHD6* induction levels, see Supporting Information Fig. S12. (c) Swollen root hairs in plants overexpressing *RSL4* on 2×MS. About half of plants have swollen root hairs on 2×MS (11 out of 28 for *35S:gRSL4‐GFP* and 14 out of 30 for *35S:RSL4‐GFP*. On the contrary, swollen root hairs on 1/2×MS were not observed from *35S:RSL4cds‐GFP* (63 seedlings) and *35S:gRSL4‐GFP* (58 seedlings). Bar, 200 μm.

For *RHD6*, *rhd6‐3* is almost hairless even under normal conditions (Menand *et al*., 2007), while the *rhd6‐3 gtl1‐1 df1‐1* mutant formed tiny root hairs with balloon‐like structures on 2×MS (Fig. 7a), suggesting that RHD6 is not responsible for the phenotype observed in *gtl1‐1 df1‐1*. In order to further investigate if overexpression of *RHD6* causes root hair swelling on 2×MS, we used an *RHD6*‐inducible overexpression line (*35S:XVE>>RHD6*), in which 5 μM 17β‐estradiol (17ED) treatment increased the level of *RHD6* mRNA by approximately 180‐fold compared to mock treatment with dimethyl sulfoxide (DMSO) (Fig. S12). Consistent with the WT phenotype, mock‐treated *35S:XVE>>RHD6* did not form root hairs on 2×MS (Fig. 7b). However, *35S:XVE>>RHD6* treated with 17ED on 2×MS produced root hairs with normal morphology, in contrast to the expectation that it might cause swollen hairs to form (Fig. 7b). These results demonstrated that the overexpression of *RHD6* in *gtl1‐1 df1‐1* does not cause the swollen‐root hair phenotype on 2×MS, but instead that *RHD6* promotes the formation of proper root hairs on 2×MS.

For *LRL3*, the *lrl3‐2* single mutant displayed a similar suppression of root hair growth on 2×MS compared to the WT (Figs 7a, S13). This is consistent with a previous finding that another single mutant for *LRL3*, *lrl3‐1*, does not affect root hair growth (Karas *et al*., 2009). Similar to *gtl1‐1 df1‐1*, *lrl3‐2 gtl1‐1 df1‐1* formed swollen root hairs on 2×MS (Fig. 7a). Therefore, expression of *LRL3* in *gtl1‐1 df1‐1* does not cause the root hair swelling in *gtl1‐1 df1‐1* on 2×MS.

In contrast to *RHD6* and *LRL3*, we found that mutation of *RSL4* rescued the *gtl1‐1 df1‐1* root hair phenotype on 2×MS. (Fig. 7a). Although the *rsl4‐1* single mutant forms root hairs under normal condition due to redundancy with *RSL2* (Shibata *et al*., 2018), both *rsl4‐1* and *rsl4‐1 gtl1‐1 df1‐1* exhibited almost no root hair growth on 2×MS. To further investigate if overexpression of *RSL4* leads to root hair swelling, we produced lines constitutively overexpressing *RSL4*, using the 35S promoter. We generated overexpression lines using either the genomic sequence (*35S:gRSL4*) or coding sequence (*35S:RSL4cds*). Both overexpression lines form only normal root hairs on 1/2×MS (Fig. 7c). On the contrary, both overexpression lines sometimes exhibited root hair swelling on 2×MS, which in contrast is never observed in the WT (Figs 4b, 7c), indicating that overexpression of *RSL4* causes this phenotype. Taken together with the phenotype of the *rsl4‐1 gtl1‐1 df1‐1* triple mutant, these data strongly suggest that higher expression of *RSL4* contributes to abnormal root hair formation on 2×MS. Notably, among the *RHD6* subfamily genes, *RSL4* was the only one which we observed to be overexpressed in *gtl1‐1 df1‐1* on 2×MS (Fig. 5).

## Discussion

### Arabidopsis plants determine root hair size by integrating multiple nutrient signals

Environmental signals affect post‐embryonic development of plants. In this study, we showed that excess nutrients, supplied in the form of 2×MS media, strongly inhibit Arabidopsis growth and have a particularly striking effect on root hairs (Fig. 1). As WT could form root hairs on 2×MS in response to exogenous IAA treatment (Fig. 3b), Arabidopsis plants actively suppress root hair growth on 2×MS. Since one of the main functions of root hairs is to enhance nutrient uptake, the root hair response to 2×MS likely reduces nutrient overloading. Our data further indicate that individual MS components do not account for the effect of 2×MS on root hair growth. Specifically, our custom MS experiments showed that neither the reduction of individual nutrients in 2×MS or their increase in 1/2×MS completely reproduces the root hair phenotypes observed on 1/2×MS or 2×MS, respectively (Figs S2, S3). These data suggest that Arabidopsis plants integrate signals transduced in response to multiple nutrients and determine an appropriate root hair response.

### Nutrient imbalance can cause root hair swelling

In addition to the strong suppression of root hair growth on 2×MS, we found that *gtl1‐1 df1‐1* on 2×MS formed swollen root hairs that exhibited reduced stiffness and often ruptured (Fig. 4). Thus, we concluded that root hairs in *gtl1‐1 df1‐1* grown on 2×MS are frail. Elongation of root hairs occurs through a highly polarized process, called tip growth. For the rapid and flexible growth response to environmental signals, factors related to tip growth are precisely organized (Braidwood *et al*., 2014; Franciosini *et al*., 2017). Indeed, many mutants have been isolated which exhibit aberrant root hair morphology (summarized in Grierson *et al*., 2014). Among the mutants that exhibit root hair morphology phenotypes, overexpression lines of ROP family genes are of particular interest. The constitutively active GTP‐bound *Rho of Plant 2* (*CA‐rop2*) or *CA‐rop11* (named *Atrac10* ^*CA*^ or *Atrop11* ^*CA*^ in the original article) induces swollen root hairs which are similar to *gtl1‐1 df1‐1* root hairs on 2×MS (Jones *et al*., 2002; Bloch *et al*., 2005). More interestingly, Bloch *et al*. (2011) reported that the emergence of swollen root hairs in *CA‐rop11* depends on NH_4_NO_3_ in the growth media. Specifically, external ammonium concentrations > 1 mM (Bloch *et al*., 2011), which is much lower than the concentration found in 1/2×MS, is required to induce root hair swelling in *CA‐rop11*. In addition, Bloch *et al*. (2011) demonstrated that NH_4_^+^/NO_3_^−^ fluxes increase the amplitude of pH oscillations at the root hair apex, which might affect cell wall properties. Thus, it was concluded from this study that when the ROP activity is upregulated by dominant mutations, the synergistic effects of pH changes and constant activation of ROP downstream effectors lead to uncontrolled cell expansion (Bloch *et al*., 2011). Indeed, we observed that reduction of N sources in 2×MS mitigated the strong suppression of root hair growth caused by growth on this medium (Fig. S3). However, 2×MS_1/2×NH_4_NO_3_ + KNO_3_ still contains 1/2×MS levels of N sources, which is far from N starvation. Given that plant growth depends just as much on the proper balance of each nutrient as it does on absolute amounts of nutrients, the strong root hair phenotype on 2×MS might be caused by nutrient imbalance rather than merely oversupply. Notably, we also found that the reduction of P in 2×MS caused root hair swelling (Fig. S2). MS medium generally contains adequate P and excess N compared to other types of plant media, such as Johnson medium (Johnson *et al*., 1957); thus, reduction of the P source should increase what is already an imbalanced P : N ratio for Arabidopsis grown on MS medium. To pinpoint the causal reason for root hair swelling, further studies on the relationship between nutrient imbalance and cell wall properties are needed.

### GTL1 and DF1 contribute to the active suppression of root hair growth in the presence of excess nutrients

In our previous study, we identified GTL1 and DF1 as suppressors of root hair growth, with the *gtl1 df1* mutant growing long root hairs on Johnson medium (Shibata *et al*., 2018), which provides a mild growth condition for Arabidopsis. In this study, we revealed another aspect of GTL1 and DF1. The *gtl1‐1 df1‐1* mutant forms frail root hairs on 2×MS (Figs 2, 3), which is a harsher growth condition for Arabidopsis (Fig. 1), showing that the activity of GTL1 and DF1 prevents formation of aberrant root hairs under certain conditions. Our AFM analysis showed that defects in GTL1 and DF1 reduce stiffness of root hairs on 2×MS compared to the WT in the same condition (Fig. 4e). Additionally, in the *gtl1‐1 df1‐1* mutant, the expression of *RHD6* subfamily genes was not properly regulated, with *RSL4* upregulated in this mutant on 2×MS, while others, namely *RSL2* and *RSL3*, were downregulated (Fig. 5). Thus, we conclude that GTL1 and DF1 function to stabilize changes in gene expression induced by variable environments, thus ensuring appropriate root hair responses. Improper gene regulation due to the lack of GTL1 and DF1 means that correct cell expansion and termination cannot be established. As overexpression of *RSL4* also caused root hair swelling on 2×MS in addition to *gtl1‐1 df1‐1*, the ROP signaling pathway likely works downstream of RSL4 and GTL1 to induce formation of abnormal root hairs in a similar manner as *CA‐rop11* discussed earlier. Furthermore, given that *RSL* genes are still expressed at detectable levels on 2×MS, other pathways are possibly also involved in the inhibition of root hair growth that do not involve the transcriptional regulation of *RSL* genes.

Notably, the expression levels of *GTL1* and *DF1* were decreased on 2×MS compared to those on 2×MS, indicating that the strong suppression of root hair growth on 2×MS is not due to induction of *GTL1* and *DF1* (Fig. S7). Induction of RSL4 activity in *Glucocorticoid Receptor:RSL4* (*GR:RSL4*)‐expressing plants showed that RSL4 induces *GTL1* expression (Vijayakumar *et al*., 2016), which points to the presence of a feedback loop from RSL4 to *GTL1* expression (Shibata & Sugimoto, 2019). Thus, it is actually unsurprising that *GTL1* and *DF1* expression levels decline together with those of *RSL2* and *RSL4* on 2×MS. However, imaging analysis demonstrated that GTL1 and DF1 protein levels are similar in early‐stage root hair cells on 1/2×MS and 2×MS (Fig. S7b). A discrepancy between mRNA and protein levels is not unusual. The synthesis and degradation of proteins are regulated independently from the transcription of their corresponding genes (Hsu *et al*., 2016). Thus, GTL1 protein stability might be regulated by post‐translational modifications like phosphorylation or SUMOylation. Notably, some publicly available proteomic data sets indicate that GTL1 undergoes post‐transactional modifications, including phosphorylation and SUMOylation (Reiland *et al*., 2009, 2011; Umezawa *et al*., 2013; Wang *et al*., 2013; Choudhary *et al*., 2015; Roitinger *et al*., 2015). In another study, it was reported that a mitogen‐activated protein (MAP) kinase, MPK4, binds GTL1, although phosphorylation of GTL1 by MPK4 was not detected (Völz *et al*., 2018). In addition, GTL1 activity is known to be regulated by calcium ion (Ca^2+^)‐dependent calmodulin (Ca^2+^/CaM). Thus, Ca^2+^/CaM binds the DNA binding domain of GTL1 and inhibits DNA binding (Weng *et al*., 2012; Yoo *et al*., 2019). Interestingly, it has also been suggested that GTL1 transcriptional repressor activity is inhibited by Ca^2+^/CaM in response to water‐deficit stress, thus leading to de‐repression of genes related to stomata development (Yoo *et al*., 2010, 2019; Weng *et al*., 2012). These data, together with the lack of transcriptional activation of *GTL1* and *DF1* on 2×MS compared to 1/2×MS, suggest the activity of GTL1 and DF1 is likely regulated by post‐translational modification(s) in the context of excess nutrients. Future studies should focus on analyzing GTL1 and DF1 protein modifications in the context of environmental responses.

### Multiple TF complexes regulate root hair growth in response to a variety of environmental conditions

We demonstrated that WT Arabidopsis plants strongly suppress root hair growth on 2×MS by repressing *RHD6* subfamily genes. Among these genes, the downregulation of *RSL2* and *RSL3* on 2×MS appears to be independent of GTL1 and DF1 *in planta* (Fig. 5), suggesting that other TFs contribute to repression of these genes. A C2H2 TF, ZINC‐FINGER‐PROTEIN1 (ZP1), was recently shown to suppress root hair growth by directly repressing *RHD6*, *RSL2* and *RSL4* (G. Han *et al*., 2020). Although this study did not investigate if ZP1 works in the context of environmental responses, it is possible that ZP1 contributes to the repression of root hair growth on 2×MS together with GTL1 and DF1. MYB DOMAIN PROTEIN 30 (MYB30) was also shown recently to negatively regulate root hair growth via direct repression of *RSL4* (Xiao *et al*., 2021). Notably, MYB30 inhibits ETHYLENE INSENSITIVE 3 (EIN3), a positive regulator of ethylene signaling, by directly interacting with this protein (Xiao *et al*., 2021). EIN3, however, is known to bind RHD6 and promotes root hair growth in response to ethylene signaling (Song *et al*., 2016b). Thus, MYB30 is expected to regulate *RSL4* competitively with EIN3. Additionally, JASMONATE‐ZIM‐DOMAIN PROTEIN (JAZ) proteins, which are key regulators of jasmonic acid (JA) signaling, also physically interact with RHD6, thus affecting root hair development in response to JA (X. Han *et al*., 2020). These data suggest that RHD6 and its binding partners work as a hub within the GRN that regulates root hair development in response to environmental signals. Moreover, our data showed that GTL1 binds to the RHD6 protein and presumably inhibits RHD6‐mediated activation of *RSL4* (Fig. 6), suggesting that GTL1 can inhibit this TF by physically interacting with it (Fig. S14). In contrast, a DF1–GL2 complex was recently reported to activate the transcription of target genes in the context of seed coat development (Xu *et al*., 2022). Thus, physical interactions between various pairs of TFs presumably enable precise and flexible regulation of root hair development in response to a variety of environmental signals.

Regarding the relationship between *RHD6* and *RSL4*, we showed that the overexpression of *RHD6* causes plants to form normal root hairs on 2×MS, while *RSL4* does not have this effect (Fig. 7b,c). Although the results are not directly comparable between plants transiently overexpressing *RHD6* and those constitutively overexpressing *RSL4*, these data imply that there might be some functional differences between these TFs. As RHD6 functions upstream of *RSL2* and probably *LRL3* in addition to *RSL4*, coordinated induction of these TFs by RHD6 in addition to *RSL4* appears to be important for proper root hair growth. Since in general plant responses to environmental signals are tightly controlled by a GRN consisting of multiple TFs (Song *et al*., 2016a; Van den Broeck *et al*., 2020), the GRN dominated by RHD6 and its binding partners likely fine tunes root hair growth in response to fluctuating environmental conditions.

### Accession numbers

Gene names, mutant names and the corresponding stock numbers are summarized in Table 1.

**Table 1:** Accession numbers.

Gene	AGI	Mutant name	Stock no.
*GTL1*	AT1G33240	*gtl1‐1*	WiscDsLox413‐416C9
*DF1*	AT1G76880	*df1‐1*	SALK_106258
*RSL4*	AT1G27740	*rsl4‐1*	GT_5_105706 (Yi *et al*., 2010)
*RHD6*	AT1G66470	*rhd6‐3*	GABI‐Kat 475E09
		*35S:XVE>>RHD6*	CS2104358
*OBP4*	AT5G60850	*obp4‐2*	SALK_085101
*OBP4*	AT5G60850	*obp4‐3*	SALKseq_108296
*GL2*	AT1G79840	*gl2‐8*	CS2106731
*LRL3*	AT5G58010	*lrl3‐2*	SALK_12380

## Author contributions

MS, DSF, BR and KS conceived the research and designed the experiments. MS and AK performed most of the experiments. RT and YH performed AFM analysis. AT performed the Co‐IP experiments. MS, DSF, YH and KS wrote the manuscript with input from all co‐authors.

## Supporting information


**Fig. S1** The expression levels of *EXPA7* and *GL2*.
**Fig. S2** The effects of series of single nutrient increase in 1/2×MS on root hair growth.
**Fig. S3** The effects of series of single nutrient reduction in 2×MS on root hair growth.
**Fig. S4** Representative images of root hair swelling at each phenotypic level.
**Fig. S5** Roots of *obp4* mutants grown on 1/2×MS or 2×MS.
**Fig. S6** Measurement of root and root hair stiffness.
**Fig. S7** Expression levels of *GTL1* and *DF1* in 1/2×MS or 2×MS media.
**Fig. S8** Protein levels of GTL1 and DF1 in 1/2×MS or 2×MS media.
**Fig. S9** Expression levels of *RSL2* and *RSL3* in *obp4* mutants.
**Fig. S10** GTL1 and DF1 suppresses *RHD6* and *RSL4* expression.
**Fig. S11** Co‐immunoprecipitation assay of RHD6 and GTL1.
**Fig. S12** The expression levels of *RHD6* and *RSL4* in corresponding overexpression lines.
**Fig. S13** Properties of the *LRL3* knockdown mutant.
**Fig. S14** A hypothetical model depicting how GTL1 and RHD6 may regulate root hair growth.

**Methods S1** Supplementary materials and methods.

**Table S1** List of PCR primers for genotyping.
**Table S2** Components of 1/2×MS, 1×MS and 2×MS media.
**Table S3** Components of the custom MS media.
**Table S4** List of PCR primers for RT‐qPCR.
**Table S5** List of PCR primers for cloning.
Please note: Wiley Blackwell are not responsible for the content or functionality of any Supporting Information supplied by the authors. Any queries (other than missing material) should be directed to the *New Phytologist* Central Office.

## Data Availability

The data that support the findings of this study are available from the corresponding author upon reasonable request.
