# Supplementary material for: Trihelix transcription factors GTL1 and DF1 prevent aberrant root hair formation in an excess nutrient condition
Source: New Phytol. 2022 Jun 17;235(4):1426–41. doi: 10.1111/nph.18255 (PMC9544051; doi:10.1111/nph.18255)
Supplement: Supplementary file 1 — Fig. S1 The expression levels of EXPA7 and GL2. Fig. S2 The effects of series of single nutrient increase in 1/2×MS on root hair growth. Fig. S3 The effects of series of single nutrient reduction in 2×MS on root hair growth. Fig. S4 Representative images of root hair swelling at each phenotypic level. Fig. S5 Roots of obp4 mutants grown on 1/2×MS or 2×MS. Fig. S6 Measurement of root and root hair stiffness. Fig. S7 Expression levels of GTL1 and DF1 in 1/2×MS or 2×MS media. Fig. S8 Protein levels of GTL1 and DF1 in 1/2×MS or 2×MS media. Fig. S9 Expression levels of RSL2 and RSL3 in obp4 mutants. Fig. S10 GTL1 and DF1 suppresses RHD6 and RSL4 expression. Fig. S11 Co‐immunoprecipitation assay of RHD6 and GTL1. Fig. S12 The expression levels of RHD6 and RSL4 in corresponding overexpression lines. Fig. S13 Properties of the LRL3 knockdown mutant. Fig. S14 A hypothetical model depicting how GTL1 and RHD6 may regulate root hair growth. [file NPH-235-1426-s003.pdf]

New Phytologist **Supporting Information**

Article title: **Trihelix transcription factors GTL1 and DF1 prevent aberrant root hair formation in an excess nutrient condition**

Article Authors: Michitaro Shibata, David S. Favero, Ryu Takebayashi, Arika Takebayashi, Ayako Kawamura, Bart Rymen, Yoichiroh Hosokawa, Keiko Sugimoto

Article acceptance date: 6 May 2022

Fig. S1. The expression levels of *EXPA7* and *GL2*

Fig. S2. The effects of series of single nutrient increase in 1/2xMS on root hair growth

Fig. S3. The effects of series of single nutrient reduction in 2xMS on root hair growth

Fig. S4. Representative images of root hair swelling at each phenotypic level

Fig. S5. Roots of *obp4* mutants grown on 1/2x or 2x MS

Fig. S6. Measurement of root and root hair stiffness

Fig. S7. Expression levels of *GTL1* and *DF1* in 1/2x or 2x MS media

Fig. S8. Protein levels of GTL1 and DF1 in 1/2x or 2x MS media

Fig. S9. Expression levels of *RSL2* and *RSL3* in *obp4* mutants

Fig. S10. GTL1 and DF1 suppresses *RHD6* and *RSL4* expression

Fig. S11. Co-immunoprecipitation assay of RHD6 and GTL1

Fig. S12. The expression levels of *RHD6* and *RSL4* in corresponding overexpression lines

Fig. S13. Properties of the *LRL3* knock-down mutant

Fig. S14. A hypothetical model depicting how GTL1 and RHD6 may regulate root hair growth

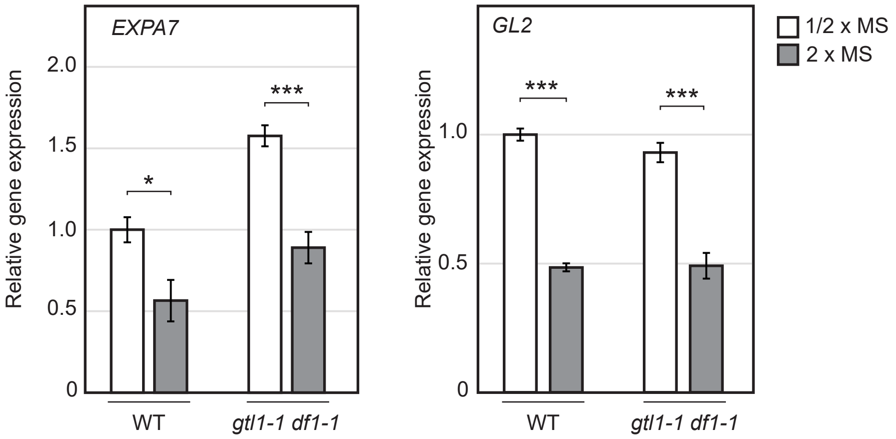

Fig. S1. The expression levels of *EXPA7* and *GL2*

RT-qPCR analysis of *EXPA7* and *GL2* in WT and *gtl1-1 df1-1* grown on 1/2x or 2x MS media. Expression levels are normalized to that of the *HEL* gene. Data are mean +/- SD. (n = 3, biological replicates). Asterisks indicate a significant difference for the same genotype grown on different types of media (Student’ s t-test, *p < 0.05, ***p < 0.001, NS = not significant).

(a)

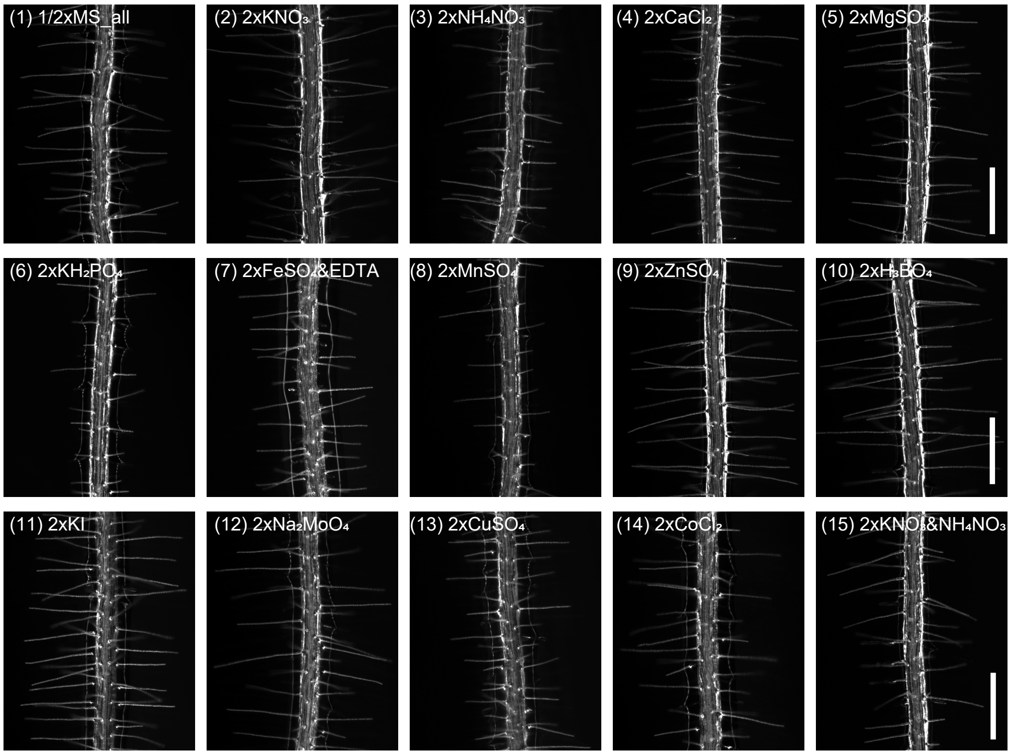

(b)

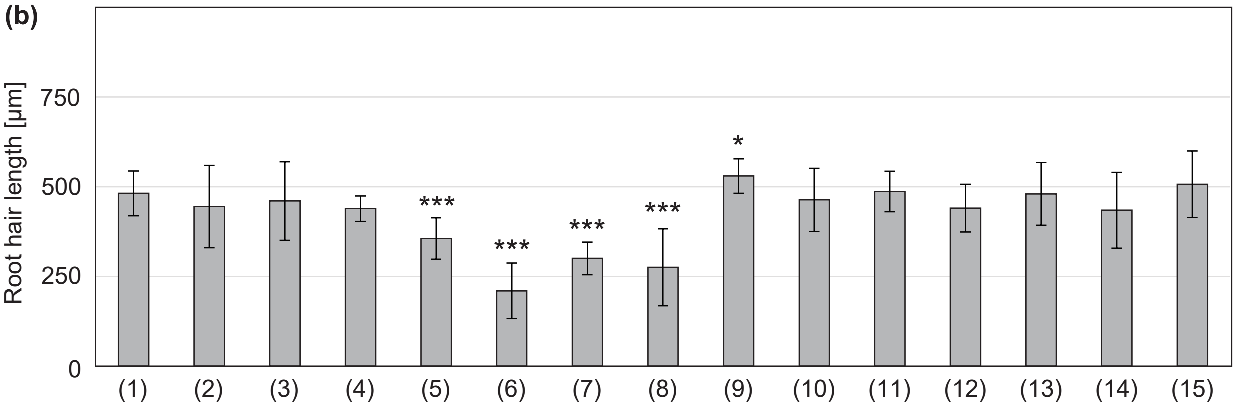

Fig. S2. The effects of series of single nutrient increase in 1/2xMS on root hair growth

(a) Images of root hairs grown on custom MS media. WT Arabidopsis were grown on 1/2xMS media for one week and then transferred to the indicated media, See Table S2 for a complete list of ingredients. Images were taken 2 days after transfer. Scale bars = 500 μm.

(b) Quantitative data for the average length of 20 longest root hairs from 12 seedlings. Asterisks indicate a significant difference compared with 1/2xMS_all (Student' s t-test, $*p < 0.05$, $***p < 0.001$).

(a)

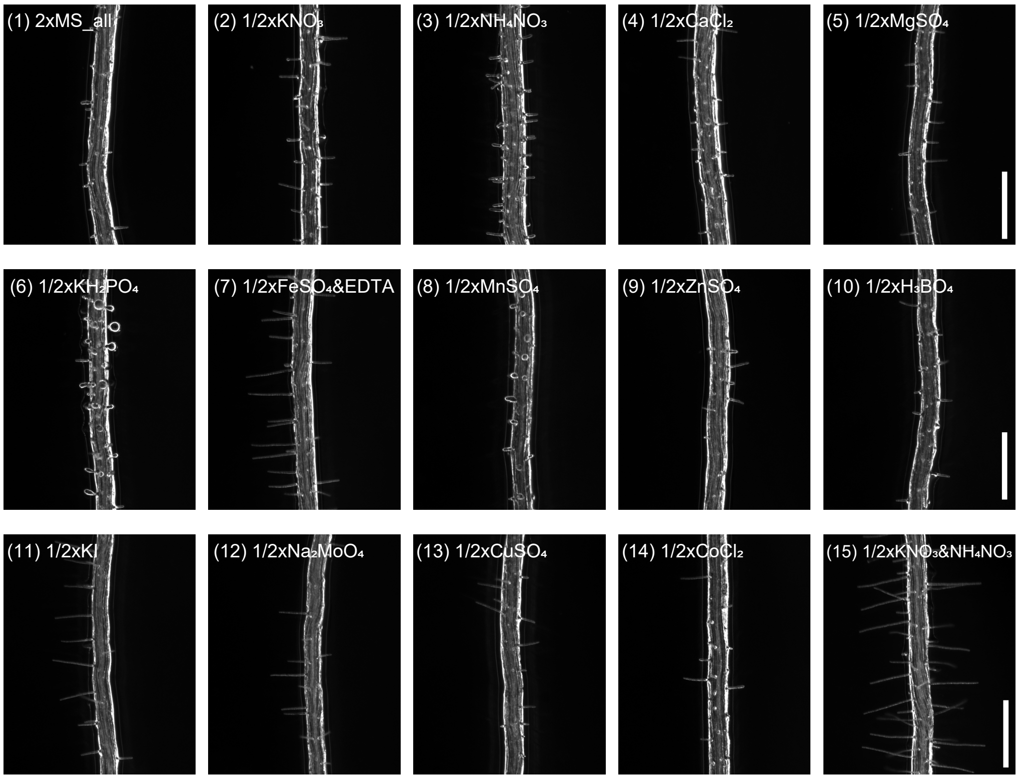

(b)

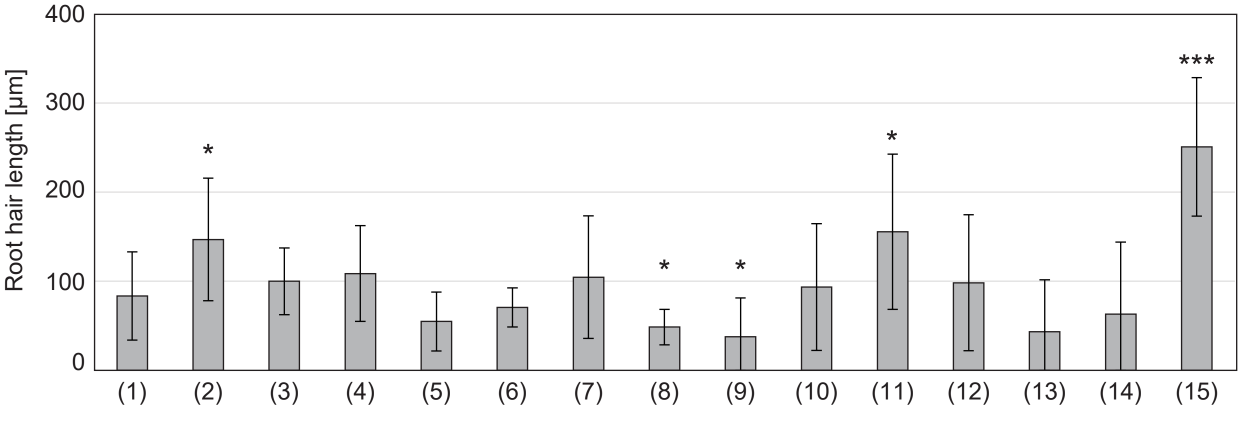

Fig. S3. The effects of series of single nutrient reduction in 2xMS on root hair growth

(a) Images of root hairs grown on custom MS media. WT Arabidopsis were grown on 1/2xMS media for one week and then transferred to the indicated media, See Table S2 for a complete list of ingredients. Images were taken 2 days after transfer. Scale bars = 500 µm.

(b) Quantitative data for the average length of 20 longest root hairs from 12 seedlings. Asterisks indicate a significant difference compared with 2xMS_all (Student' s t-test, $*p < 0.05$, $***p < 0.001$).

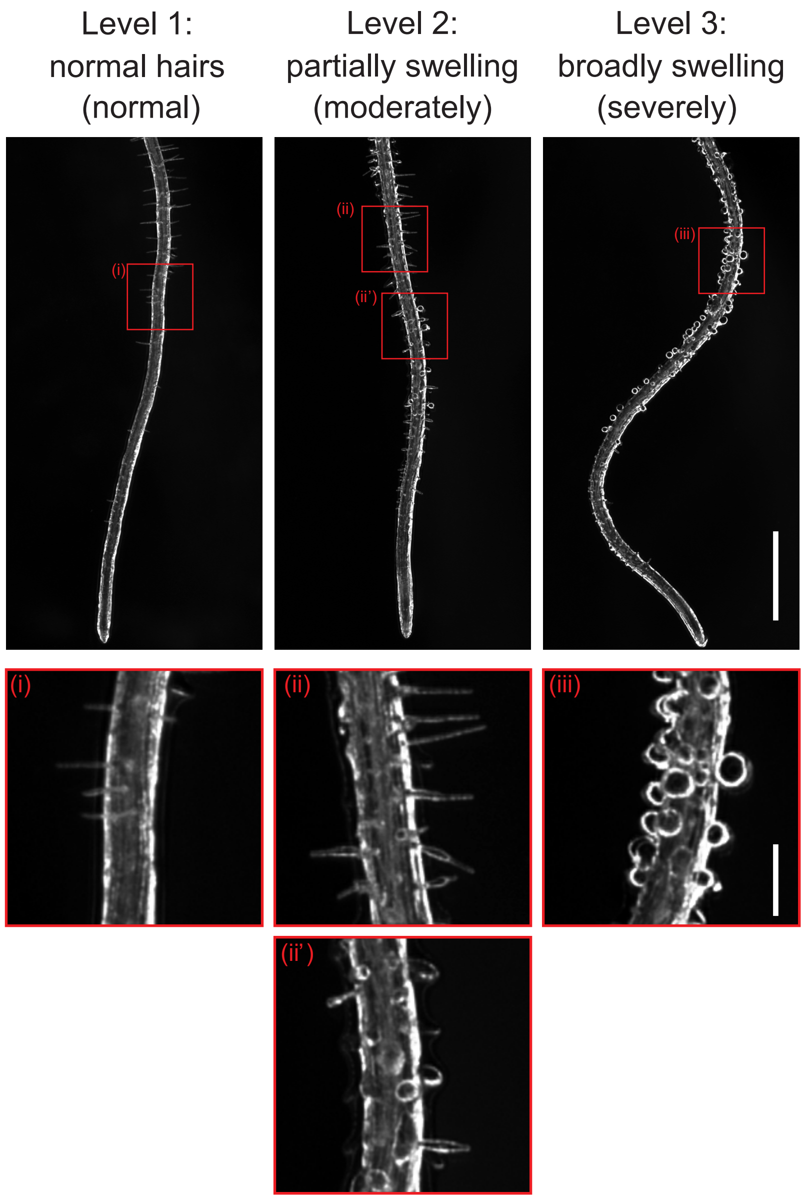

Fig. S4. Representative images of root hair swelling at each phenotypic level

Roots with no visible root hairs or root hairs with tube-like structure are categorized as Levels 1. Roots with partially swollen root hairs are categorized as Level 2. Roots with broad swelling are categorized as Level 3. Scale bars = 500 μm for the upper panel and 100 μm for the lower panel.

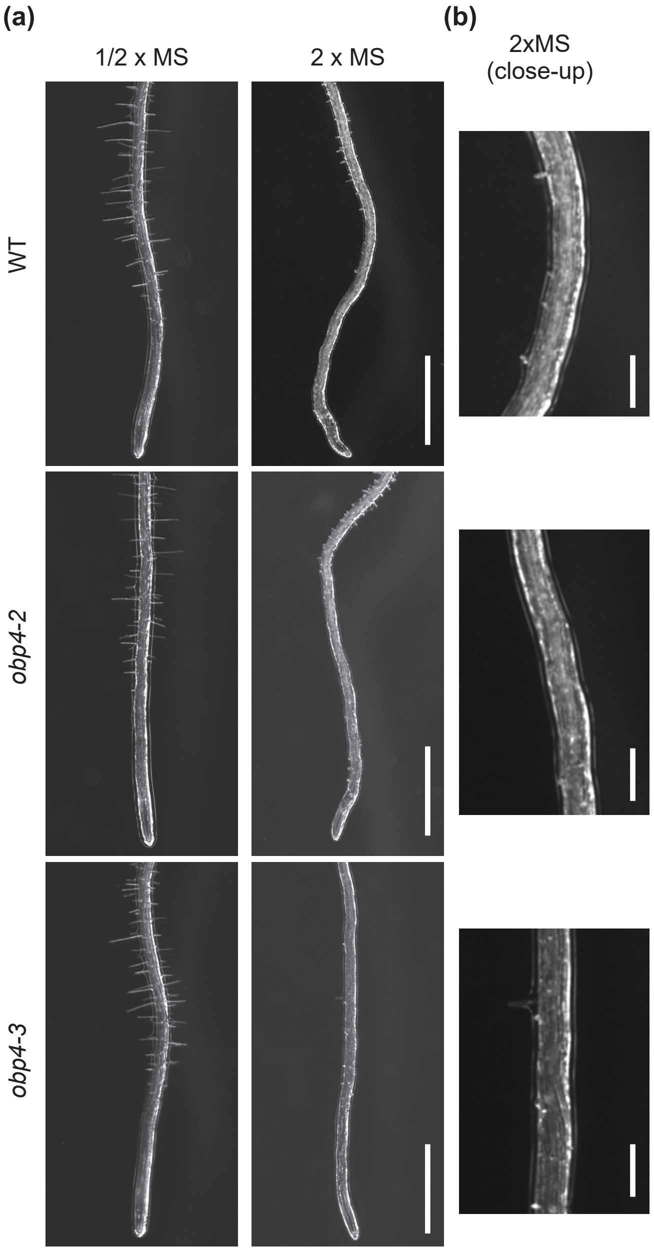

Fig. S5. Roots of *obp4* mutants grown on 1/2x or 2x MS

(a) Images of root tips of the WT, *obp4-2* and *obp4-3*, on 1/2x or 2x MS media and close-up images of roots on 2xMS in (b). Both *obp4* mutants show similar responses to the WT on 2xMS. Scale bars = 1000 μm in (a) and 200 μm in (b).

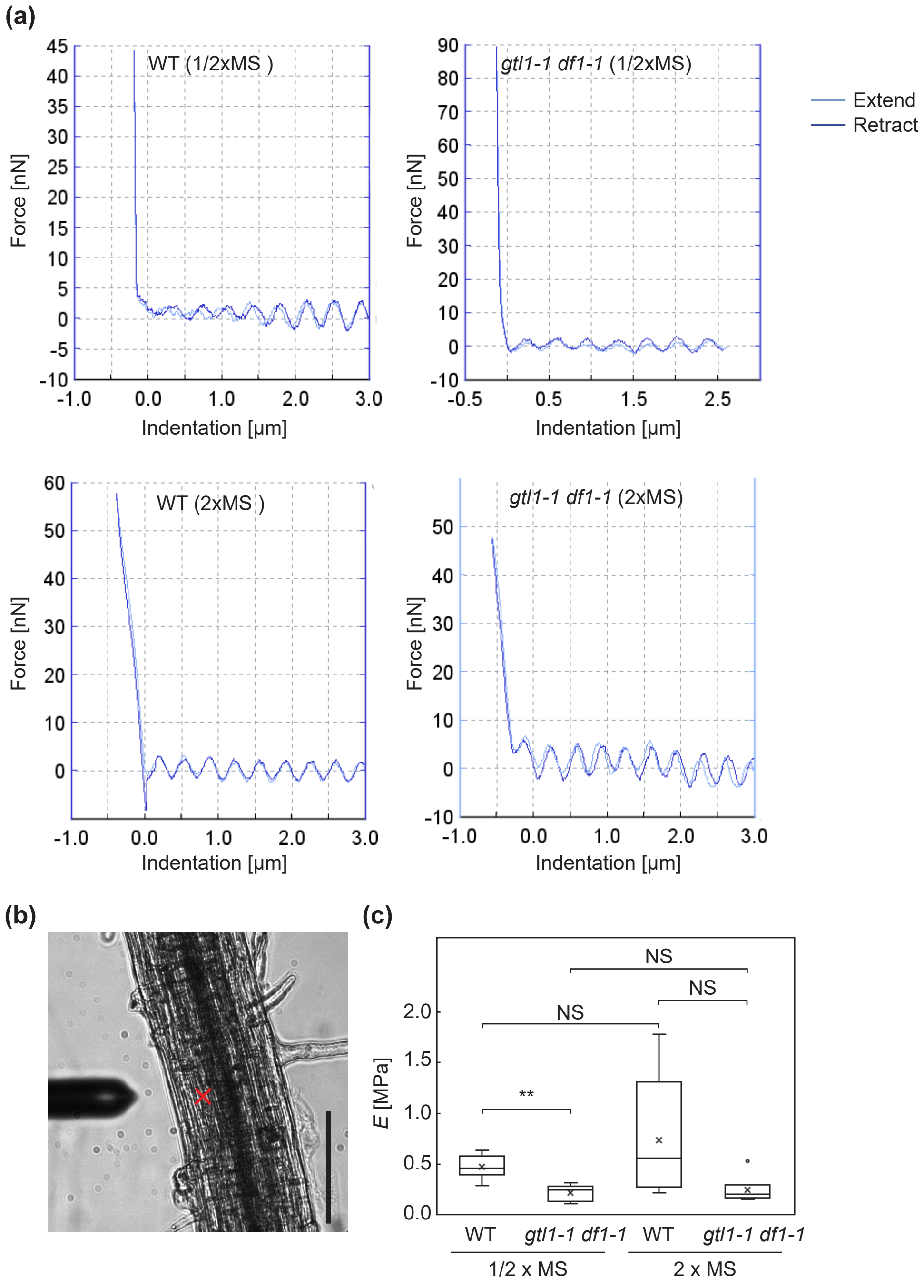

Fig. S6. Measurement of root and root hair stiffness

(a) Force-indentation curves from the AFM measurements. Representative results are shown for each phenotype and condition combination. Processed data are shown in Fig 4c.

(b) A microscopic image of a root used for AFM analysis. “x” in the image indicates the contact point of the probe. Note that the analyzed cell is not the indicated cell because the probe contacts the sample from the top, while the image was taken from the bottom by an inverted microscope. Scale bar = 100 μm.

(c) Box plots showing primary root stiffness scores, as determined from analysis of the force indentation curves. The boxes show the lower quartile and upper quartile values. The whiskers show the maximum and minimum values that are still within a factor of 1.5 times the interquartile range beyond each quartile. The horizontal bar and the cross in each box indicate the median and mean value, respectively. Y axis indicates Young's modulus (E). Asterisks indicate a significant difference (Student’ s t-test, *$p < 0.05$, **$p < 0.01$, NS = not significant).

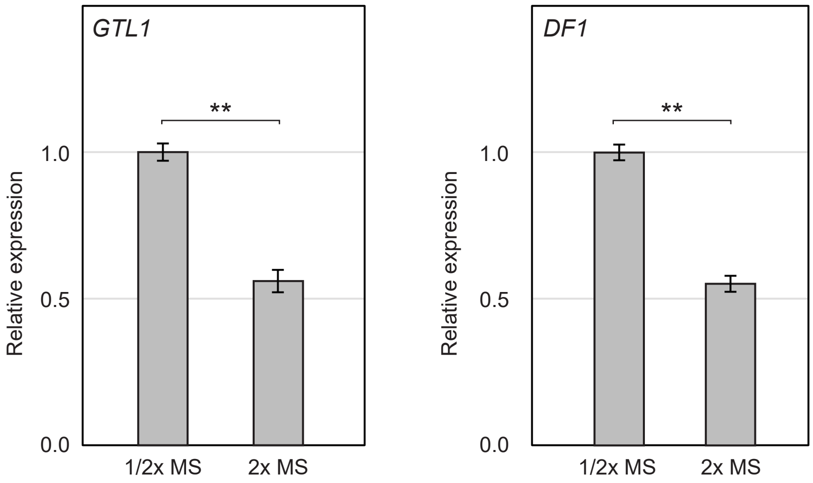

Fig. S7. Expression levels of *GTL1* and *DF1*

RT-qPCR analysis of GTL1 and DF1 grown on 1/2x or 2x MS media. Expression levels are normalized to that of the *HEL* gene. Data are mean +/- SD. (n = 3, biological replicates). Asterisks indicate a significant difference (Student' s t-test, **p < 0.01).

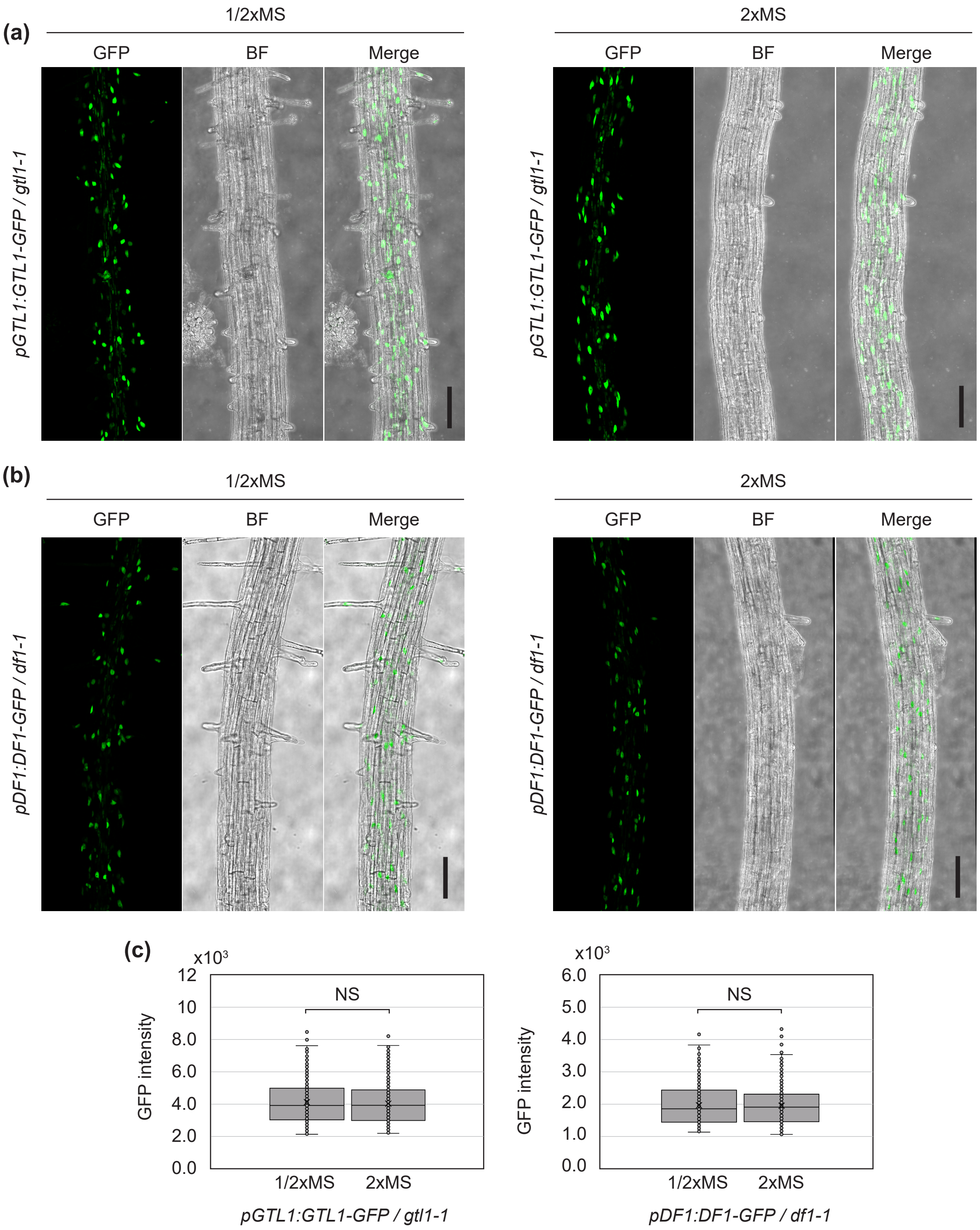

Fig. S8. Protein levels of GTL1 and DF1 in 1/2x or 2x MS media

(a, b) The confocal images of *pGTL1:GTL1-GFP / gtl1-1* (a) and *pDF1:DF-GFP / df1-1* (b) grown on 1/2xMS (left) or 2xMS (right). Each panel indicate GFP, bright field (BF) and the merged images, respectively. Scale Bars = 100 μm

(c) Quantification of GFP intensity per nucleus. Early stages of root epidermal cells were used for the quantification. The boxes show the lower quartile and upper quartile values. The whiskers show the maximum and minimum values that are still within a factor of 1.5 times the interquartile range beyond each quartile. The horizontal bar and the cross in each box indicate the median and mean value, respectively. Each dot in the box plot indicates the mean value of GFP intensity from a single nucleus. NS indicates not significant (student' s t-test, n = 464, 800, 332, 392 for GTL1-GFP on 1/2xMS, GTL1-GFP on 2xMS, DF1-GFP on 1/2xMS, DF1-GFP on 2xMS, respectively.

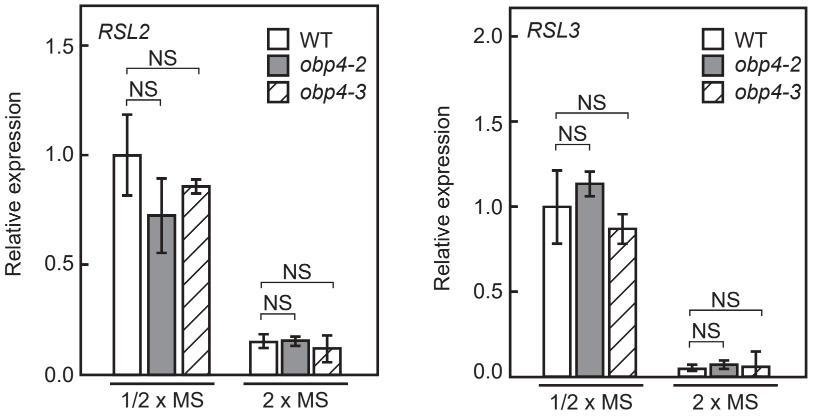

Fig. S9. Expression levels of *RSL2* and *RSL3* in *obp4* mutants

RT-qPCR analysis of *RSL2* and *RSL3* grown on 1/2x or 2x MS media. Expression levels are normalized to that of the *HEL* gene and shown as relative values compared to WT grown on 1/2xMS. Data are mean +/- SD. (n = 3, biological replicates). NS indicates not significant (Student’ s t-test).

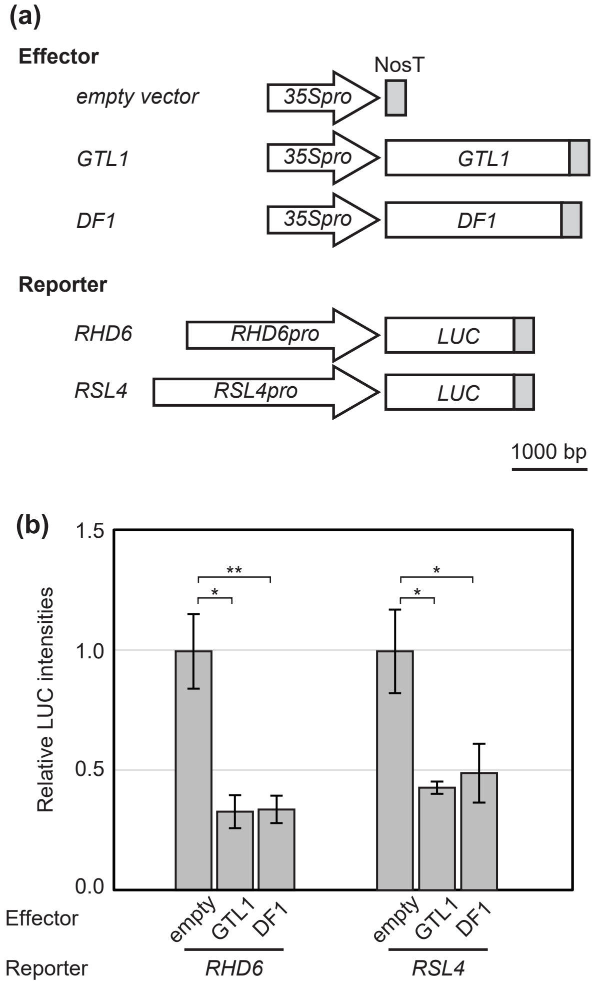

Fig. S10. GTL1 and DF1 suppresses *RHD6* and *RSL4* expression

(a) Constructs used for the promoter-Luciferase assay. The arrow-shaped boxes indicate promoters, open and closed boxes indicate coding region and terminator, respectively. Scale bar = 1000 bp.

(b) Results from the promoter-Luciferase assay using MM2d culture cells. GTL1 was used as the effector. *RHD6* and *RSL4* promoter sequences fused with Luciferase were used as reporters. Data are mean +/- SD (n = 3). Asterisks indicate significant difference compared to vector control (Student' s t-test, *p < 0.05, **p < 0.01).

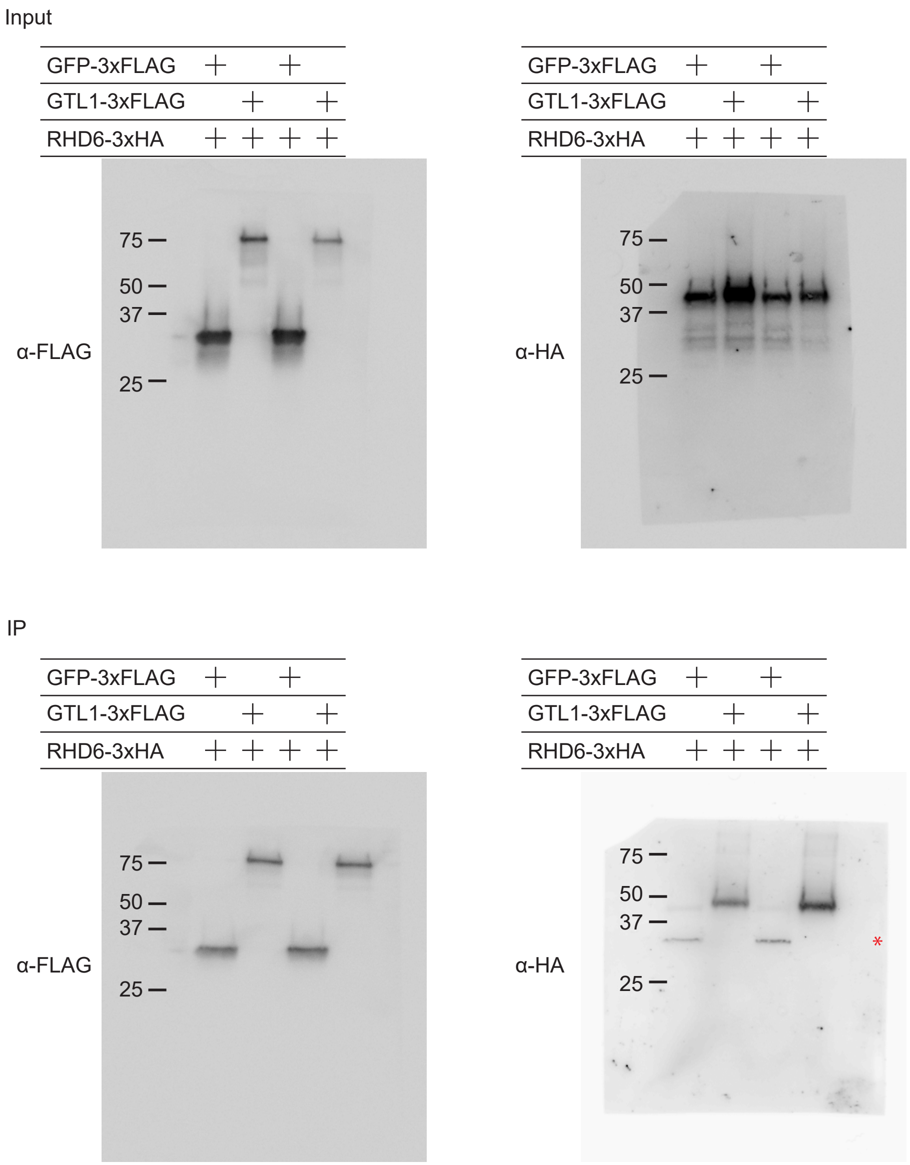

Fig. S11. Co-immunoprecipitation assay of RHD6 and GTL1

The unedited membrane images used for Fig. 6e. The predicted protein sizes are 26.6 kDa for GFP-3xFLAG, 76.9 kDa for GTL1-3xFLAG and 34.8 kDa for RHD6-3xHA. The protein ladder is shown on the left side of each membrane. The numbers indicate the protein size (kDa). The asterisk indicates a non-specific band. Two biological duplicates are shown in a single membrane.

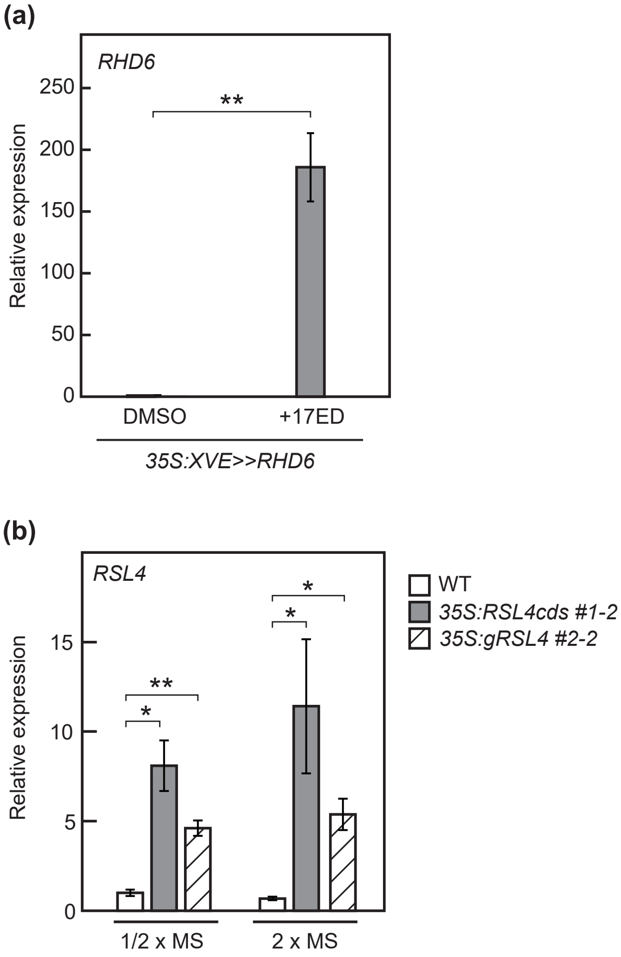

Fig. S12. The expression levels of *RHD6* and *RSL4* in corresponding overexpression lines

(a) RT-qPCR analysis of *RHD6* in *35S:XVE>>RHD6*. Total RNA was purified after 24h of treatment with 10μM 17ED. DMSO was used as a control treatment. Expression levels are normalized to that of the *UBQ10* gene. Data are mean +/- SD. (n = 3, biological replicates). Asterisks indicate a significant difference compared to the control condition (Student' s t-test, *p < 0.05, **p < 0.01, ***p < 0.001, NS = not significant).

(b) RT-qPCR analysis of *RSL4* in *35S:RSL4cds #1-2* and *35S:gRSL4 #2-2*. Expression levels are normalized to that of the *UBQ10* gene. Data are mean +/- SD. (n = 3, biological replicates). Asterisks indicate a significant difference compared to the WT grown on the same type of medium (Student' s t-test, *p < 0.05, **p < 0.01).

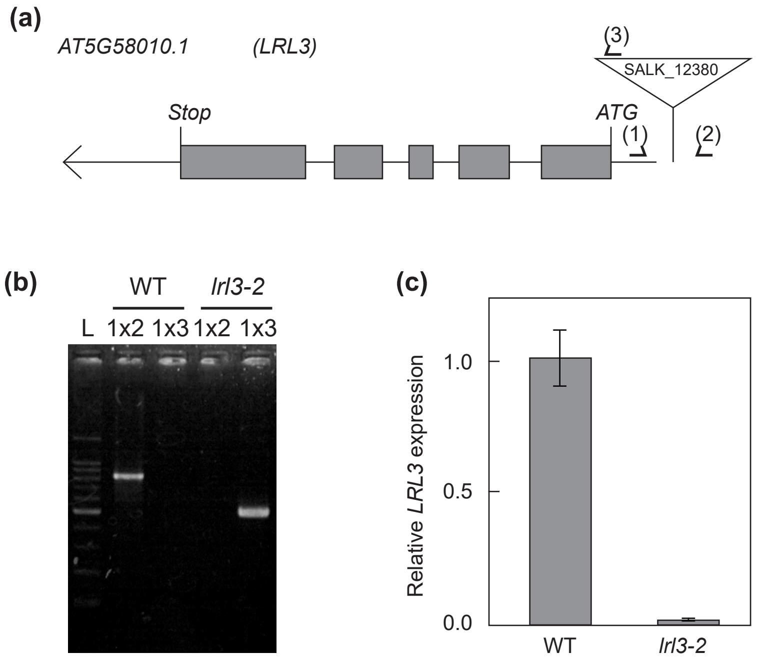

Fig. S13. Properties of the *LRL3* knock-down mutant

(a) Gene structure of *AT5G58010*/*LRL3*. Closed boxes denote exons. The triangle represents the position of the T-DNA insertion (SALK_012380/*lrl3-2*). Arrows indicate primers used for genotyping.

(b) Image of an agarose gel showing PCR-amplified DNA fragments for genotyping. Primer sets are shown above each lane. L indicates 100bp Ladder.

(c) Relative expression level of *LRL3* in the *lrl3-2* mutant. Data are mean +/- SD. (n = 3, biological replicates).

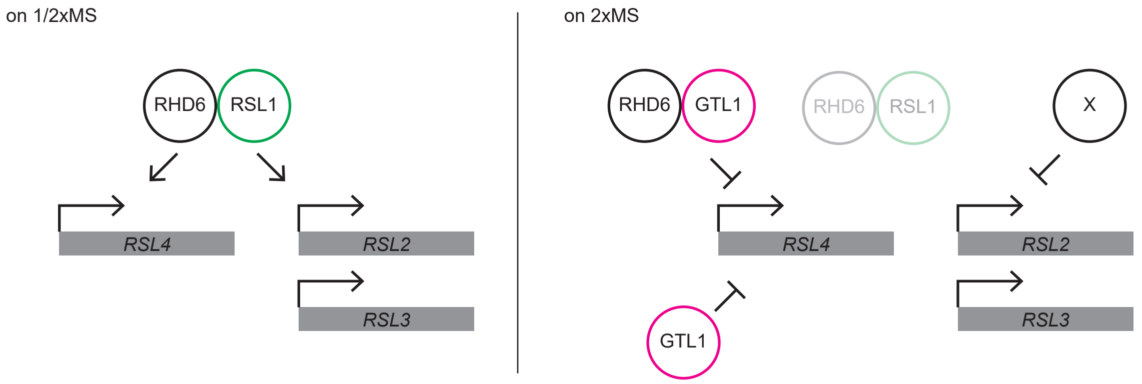

Fig. S14 A hypothetical model depicting how GTL1 and RHD6 may regulate root hair growth

On 1/2xMS, RHD6 acting together with RSL1 induces the expression of downstream genes in a developmentally programed manner. On 2xMS, GTL1 and RHD6 form a protein complex, thus reducing downstream gene expression. In parallel, GTL1 represses *RSL4* expression by binding to a different promoter region from the region which RHD6 binds to; thus, GTL1 may also suppress root hair growth independently from RHD6. The expression of *RSL2* and *RSL3* is repressed by an unidentified factor "X" , which is presumably another key braking system for root hair growth. Coordinated induction of *RSL4* together with other genes like *RSL2* and *RSL3* is likely necessary for root hairs to grow normally. The normal and blunt ended arrows indicate activation and suppression, respectively.
