# Supplementary material for: Trihelix transcription factors GTL1 and DF1 prevent aberrant root hair formation in an excess nutrient condition
Source: New Phytol. 2022 Jun 17;235(4):1426–41. doi: 10.1111/nph.18255 (PMC9544051; doi:10.1111/nph.18255)
Supplement: Supplementary file 2 — Methods S1 Supplementary materials and methods. [file NPH-235-1426-s002.pdf]

New Phytologist **Supporting Information**

Article title: **Trihelix transcription factors GTL1 and DF1 prevent aberrant root hair formation in an excess nutrient condition**

Article Authors: Michitaro Shibata, David S. Favero, Ryu Takebayashi, Arika Takebayashi, Ayako Kawamura, Bart Rymen, Yoichiroh Hosokawa, Keiko Sugimoto

Article acceptance date: 6 May 2022

Methods S1. Supplementary Materials and Methods

Quantification of GFP intensity

For GTL1-GFP and DF1-GFP proteins, light of wavelength 488 nm from an argon-ion laser was used for the excitation and emitted light of wavelengths from 500-600 nm were obtained. Bright field images were concurrently recorded with the corresponding confocal images. The root images were produced from tiling and z-stack images with "mosaic merge" and "maximum projection" using LAS-X software (version 3.7.2.22383; Leica). For quantification of GFP intensity, images were exported as 16-bit tiff-format files and the intensity of fluorescence in nuclei measured using "Analyze Particles" combined with "Threshold (Otsu)". The mean fluorescence intensity within individual nuclei was used for quantification.

Plasmid construction and plant transformation

For *35S:RSL4gDNA-GFP* and *35S:RSL4cDNA-GFP*, the coding regions of *RSL4* (AT1G27740.1) were amplified by PCR (PrimeStar max, Takara) using genomic DNA and cDNA as templates, respectively. The PCR products were introduced into pDONR221 (Invitrogen) via BP reactions and subsequently transferred to the *pGWB505* destination vector (Nakagawa *et al.*, 2007) via LR reactions. The resulting constructs were then introduced into Arabidopsis plants via the floral dip method (Clough & Bent, 1998). A set of primers used for PCR amplification is provided in Table S5.

Promoter-luciferase assay

The effector vectors *35S:GTL1* and *35S:RHD6* were previously described in Shibata *et al.*, 2018 and Rymen *et al.* 2017, respectively. *35S:DF1* was prepared for this study. Briefly, the genomic sequence of *DF1* was amplified by PCR (PrimeSTAR max, Takara) and cloned into the 35S:SG plasmid (Mitsuda *et al.*, 2005) by ligation (Takara). For the reporter vectors, the promoter regions were amplified by PCR (PrimeSTAR max, Takara) and cloned into pGEM/T-EASY containing LUC+ (Ohta *et al.*, 2001) for *RSL4,* and R4L1pUGW35 (Nakamura *et al.*, 2009) for *RHD6*. The promoter truncation series was prepared by inverse PCR. Primers used for the cloning are shown in Table S5. As an internal control, the *pPTRL* vector, which drives the expression of a *Renilla LUC* gene under the control of the *CaMV p35S* promoter, was used.

Co-Immunoprecipitation (Co-IP)

Full-length cDNA of *EGFP*, *GTL1* or *RHD6* was fused with *3xFLAG, 3xFLAG* or *3xHA* by PCR, respectively. Primers used for the PCR are shown in Table S5. The resulting PCR products containing an affinity tag were individually cloned into pDONR207 (Invitrogen) *via* BP reactions and subsequently transferred into pEAQ-HT-DEST1 (Sainsbury *et al.*, 2009) *via* LR reactions. The resulting plasmids, *EGFP-3xFLAG* or *GTL-3xFLAG*, were co-injected with *RHD6-3xHA* into tobacco leaves by agroinfiltration. The infected leaves were incubated for 3 days, harvested and immediately frozen with liquid nitrogen. The following co-immunoprecipitation assay was performed as described previously with some modifications (Kadota *et al.*, 2016). Briefly, the harvested samples were ground using a Multi-beads shocker (Yasui Kikai) using the settings 3000 Hz, 15 sec x3, and subsequently soaked in an extraction buffer containing 50 mM Tris-HCl, pH 7.5, 150 mM NaCl, 10 % glycerol, 5 mM DTT, 2.5 mM NaF, 1 mM $Na_2MoO_4 \cdot 2H_2O$, 0.5 % (w/v) polyvinylpyrrolidone, an appropriate amount of cOmplete Protease Inhibitor Cocktail (Roche), 100 μM phenylmethylsulphonyl fluoride and 2 % (v/v) IGEPAL CA-630 (Sigma-Aldrich). The protein extract was homogenized with the Branson Digital Sonifier 450D (Branson) using the settings 50%, 10sec x1. EGFP-3xFLAG and RHD6-3xFLAG were immunoprecipitated using anti-DYKDDDDK MicroBeads (miltenyi biotec) and a μMACS separator (miltenyi biotec). The input and immunoprecipitated proteins were analyzed via western blot using either an anti-FLAG (Cat. No. A8592, Sigma-Aldrich) or an anti-HA (Cat. No.12 013 819 001, Roche) HRP-conjugated antibody used at a working concentration of 1:4000 or 1:2500, respectively. Amersham ECL Select Western Blotting Detection Reagent (cytiva) was used for signal detection.

Reference

Clough SJ, Bent AF. **1998**. Floral dip: a simplified method for Agrobacterium -mediated transformation of Arabidopsis thaliana. *The Plant Journal* **16**: 735–743.

Kadota Y, Macho AP, Zipfel C. **2016**. Immunoprecipitation of Plasma Membrane Receptor-Like Kinases for Identification of Phosphorylation Sites and Associated Proteins. *Methods in Molecular Biology (Clifton, N.J.)* **1363**: 133–144.

Mitsuda N, Seki M, Shinozaki K, Ohme-Takagi M. **2005**. The NAC Transcription Factors NST1 and NST2 of Arabidopsis Regulate Secondary Wall Thickenings and Are Required for Anther Dehiscence. *The Plant Cell* **17**: 2993–3006.

Nakagawa T, Suzuki T, Murata S, Nakamura S, Hino T, Maeo K, Tabata R, Kawai T, Tanaka K, Niwa Y, *et al.* 2007. Improved Gateway binary vectors: high-performance vectors for creation of fusion constructs in transgenic analysis of plants. *Bioscience, Biotechnology, and Biochemistry* **71**: 2095–2100.

Nakamura S, Nakano A, Kawamukai M, Kimura T, Ishiguro S, Nakagawa T. **2009**. Development of Gateway Binary Vectors, R4L1pGWBs, for Promoter Analysis in Higher Plants. *Bioscience, Biotechnology, and Biochemistry* **73**: 2556–2559.

Ohta M, Matsui K, Hiratsu K, Shinshi H, Ohme-Takagi M. **2001**. Repression domains of class II ERF transcriptional repressors share an essential motif for active repression. *The Plant Cell* **13**: 1959–1968.

Rymen B, Kawamura A, Schäfer S, Breuer C, Iwase A, Shibata M, Ikeda M, Mitsuda N, Koncz C, Ohme-Takagi M, *et al.* 2017. ABA Suppresses Root Hair Growth via the OBP4 Transcriptional Regulator. *Plant Physiology* **173**: 1750–1762.

Sainsbury F, Thuenemann EC, Lomonossoff GP. **2009**. pEAQ: versatile expression vectors for easy and quick transient expression of heterologous proteins in plants. *Plant Biotechnology Journal* **7**: 682–693.

Shibata M, Breuer C, Kawamura A, Clark NM, Rymen B, Braidwood L, Morohashi K, Busch W, Benfey PN, Sozzani R, *et al.* 2018. GTL1 and DF1 regulate root hair growth through transcriptional repression of ROOT HAIR DEFECTIVE 6-LIKE 4 in Arabidopsis. *Development* **145**. dev159707
