# Supplementary material for: Trihelix transcription factors GTL1 and DF1 prevent aberrant root hair formation in an excess nutrient condition
Source: New Phytol. 2022 Jun 17;235(4):1426–41. doi: 10.1111/nph.18255 (PMC9544051; doi:10.1111/nph.18255)
Supplement: Supplementary file 3 — Table S1 List of PCR primers for genotyping. Table S2 Components of 1/2×MS, 1×MS and 2×MS media. Table S3 Components of the custom MS media. Table S4 List of PCR primers for RT‐qPCR. Table S5 List of PCR primers for cloning. Please note: Wiley Blackwell are not responsible for the content or functionality of any Supporting Information supplied by the authors. Any queries (other than missing material) should be directed to the New Phytologist Central Office. [file NPH-235-1426-s001.pdf]

New Phytologist **Supporting Information**

Article title: **Trihelix transcription factors GTL1 and DF1 prevent aberrant root hair formation in an excess nutrient condition**

Article Authors: Michitaro Shibata, David S. Favero, Ryu Takebayashi, Arika Takebayashi, Ayako Kawamura, Bart Rymen, Yoichiroh Hosokawa, Keiko Sugimoto

Article acceptance date: 6 May 2022

Table S1. List of PCR Primers for genotyping
Table S2. Components of 1/2x, 1x and 2x MS media
Table S3. Components of the custom MS media
Table S4. List of PCR Primers for RT-qPCR
Table S5. List of PCR Primers for Cloning

Table S1. List of PCR Primers for Genotyping

mutant name	Accettion	Primer name	sequence	For T-DNA	Reference
gtl1-1	WiscDsLox413-416C9	*WF627-F1*	TTCTCGTCTCATAGCTCATCG	627-R1 x Wis_pDS-LOX-LB	Breuer et al 2009
		WF627-R1	TGGCCATCTTGATGATGATGG		Breuer et al 2009
		Wis_pDS-LOX-LB	GGGTTTCGCTCATGTGTTGA		This study
df1-1	SALK_106258	DF1_258-F1	TGCTGATTGATCCACTTCTCA	258R5 x LBa1	Shibata et al 2018
		DF1_258 R5	GAGATGTTTGGAAACGAAGGTAC		Shibata et al 2018
		LBa1	GCGTGGACCGCTTGCTGCAACT		-
obp4-2	SALKseq_085101	obp4-2 LP	TCAAGCAACGTAGTTCAATGG	LBb1.3 x RP	Rymen et al., 2017
		obp4-2 RP	GTTCTTGCAAAAGTGACGAGG		Rymen et al., 2017
		LBb1.3	ATTTTGCCGATTTCGGAAC		-
obp4-3	SALKseq_108296	obp4-3 LP	CCGTTTTAAACCCATCAAATAAAA	LBb1.3 x RP	Rymen et al., 2017
		obp4-3 RP	TTTACGGTAACGGGATCGAG		Rymen et al., 2017
		LBb1.3	ATTTTGCCGATTTCGGAAC		-
rhd6-3	GABI-Kat 475E09	rhd6 F	GTTCCCAATGGCACCAAGGTACA	rhd6 F x GABI LB	Menand et al 2007
		rhd6 R	TAACTTGAGAAATGTCAGGAGC		This study
		GABI LB	CCCATTTGGACGTGAATGTAGACAC		Menand et al 2007
rsl4-1	GT_5_105706	rsl4-1 homozygote F	GAAAGCTTCGGTCACAAGTGTTAAA	JIC-RB1 x rsl4-1 LB R	Yi et al 2010
		rsl4-1 LB R	TTGTAAGCCAATGGTGCGTACAT		Yi et al 2010
		JIC-RB1	CCGAACAAAAATACCGGTTCCC		Yi et al 2010
lrl3-2	SALK_012380	lrl3_380_LP2	CGAGCAAGCCGAAAACGATA	LBa1 x RP2	This study
		lrl3_380_RP2	CTCTGTTCCCTCCCCTGTTT		This study
		LBa1	GCGTGGACCGCTTGCTGCAACT		-

Table S2. Components of 1/2x, 1x and 2x MS media

chemicals	1/2xMS [mg/l]	1xMS [mg/l]	2xMS [mg/l]
KNO_3	950	1900	3800
NH_4NO_3	825	1650	3300
$CaCl_2 \cdot 2H_2O$	220	440	880
$MgSO_4 \cdot 7H_2O$	185	370	740
KH_2PO_4	85	170	340
2NaEDTA $2H_2O$	18.65	37.3	74.6
$FeSO_4 \cdot 7H_2O$	13.9	27.8	55.6
MnSO4·$5H_2O$	12.05	24.1	48.2
$ZnSO_4 \cdot 7H_2O$	4.3	8.6	17.2
H_3BO_3	3.1	6.2	12.4
KI	0.415	0.83	1.66
$Na_2MoO_4 \cdot 2H_2O$	0.125	0.25	0.5
$CuSO_4 \cdot 5H_2O$	0.0125	0.025	0.05
$CoCl_2 \cdot 6H_2O$	0.0125	0.025	0.05
sucrose (g)	10	10	10
250mM MES pH5.7 (ml)	10	10	10
55mM myo-inositol (µl)	1000	1000	1000
gellan gum (g)	6	6	6

Table S3. Components of the custom MS media

	omission of nutrient composition from 2xMS media														
		KNO_3	NH_4NO_3	$CaCl_2{\cdot}2H_2O$	$MgSO4{\cdot}7H_2O$	KH_2PO_4	2NaEDTA $2H_2O$	$FeSO_4{\cdot}7H_2O$	$MnSO4{\cdot}5H_2O$	$ZnSO_4{\cdot}7H_2O$	H_3BO_3	KI	$Na_2MoO_4{\cdot}2H_2O$	$CuSO4{\cdot}5H_2O$	$CoCl_2{\cdot}6H_2O$
1	2xMS_All	2x	2x	2x	2x	2x	2x	2x	2x	2x	2x	2x	2x	2x	2x
2	2xMS_1/2xKNO_3	1/2x	2x	2x	2x	2x	2x	2x	2x	2x	2x	2x	2x	2x	2x
3	2xMS_1/2xNH_4NO_3	2x	1/2x	2x	2x	2x	2x	2x	2x	2x	2x	2x	2x	2x	2x
4	2xMS_1/2x$CaCl_2$	2x	2x	1/2x	2x	2x	2x	2x	2x	2x	2x	2x	2x	2x	2x
5	2xMS_1/2x$MgSO_4$	2x	2x	2x	1/2x	2x	2x	2x	2x	2x	2x	2x	2x	2x	2x
6	2xMS_1/2xKH_2PO_4	2x	2x	2x	2x	1/2x	2x	2x	2x	2x	2x	2x	2x	2x	2x
7	2xMS_1/2x$FeSO_4$ 1/2xEDTA	2x	2x	2x	2x	2x	1/2x	1/2x	2x	2x	2x	2x	2x	2x	2x
8	2xMS_1/2x$MnSO_4$	2x	2x	2x	2x	2x	2x	2x	1/2x	2x	2x	2x	2x	2x	2x
9	2xMS_1/2x$ZnSO_4$	2x	2x	2x	2x	2x	2x	2x	2x	1/2x	2x	2x	2x	2x	2x
10	2xMS_1/2xH_3BO_3	2x	2x	2x	2x	2x	2x	2x	2x	2x	1/2x	2x	2x	2x	2x
11	2xMS_1/2xKI	2x	2x	2x	2x	2x	2x	2x	2x	2x	2x	1/2x	2x	2x	2x
12	2xMS_1/2xNa_2MoO_4	2x	2x	2x	2x	2x	2x	2x	2x	2x	2x	2x	1/2x	2x	2x
13	2xMS_1/2x$CuSO_4$	2x	2x	2x	2x	2x	2x	2x	2x	2x	2x	2x	2x	1/2x	2x
14	2xMS_1/2x$CoCl_2$	2x	2x	2x	2x	2x	2x	2x	2x	2x	2x	2x	2x	2x	1/2x
15	2xMS_1/2xKNO_3_1/2xNH_4NO_3	1/2x	1/2x	2x	2x	2x	2x	2x	2x	2x	2x	2x	2x	2x	2x

	addition of nutrient composition from 1/2xMS media														
		KNO_3	NH_4NO_3	$CaCl_2{\cdot}2H_2O$	$MgSO4{\cdot}7H_2O$	KH_2PO_4	2NaEDTA $2H_2O$	$FeSO_4{\cdot}7H_2O$	$MnSO4{\cdot}5H_2O$	$ZnSO_4{\cdot}7H_2O$	H_3BO_3	KI	$Na_2MoO_4{\cdot}2H_2O$	$CuSO4{\cdot}5H_2O$	$CoCl_2{\cdot}6H_2O$
1	1/2xMS_All	1/2x	1/2x	1/2x	1/2x	1/2x	1/2x	1/2x	1/2x	1/2x	1/2x	1/2x	1/2x	1/2x	1/2x
2	1/2xMS_2xKNO_3	2x	1/2x	1/2x	1/2x	1/2x	1/2x	1/2x	1/2x	1/2x	1/2x	1/2x	1/2x	1/2x	1/2x
3	1/2xMS_2xNH_4NO_3	1/2x	2x	1/2x	1/2x	1/2x	1/2x	1/2x	1/2x	1/2x	1/2x	1/2x	1/2x	1/2x	1/2x
4	1/2xMS_2x$CaCl_2$	1/2x	1/2x	2x	1/2x	1/2x	1/2x	1/2x	1/2x	1/2x	1/2x	1/2x	1/2x	1/2x	1/2x
5	1/2xMS_2x$MgSO_4$	1/2x	1/2x	1/2x	2x	1/2x	1/2x	1/2x	1/2x	1/2x	1/2x	1/2x	1/2x	1/2x	1/2x
6	1/2xMS_2xKH_2PO_4	1/2x	1/2x	1/2x	1/2x	2x	1/2x	1/2x	1/2x	1/2x	1/2x	1/2x	1/2x	1/2x	1/2x
7	1/2xMS_2x$FeSO_4$ 2xEDTA	1/2x	1/2x	1/2x	1/2x	1/2x	2x	2x	1/2x	1/2x	1/2x	1/2x	1/2x	1/2x	1/2x
8	1/2xMS_2x$MnSO_4$	1/2x	1/2x	1/2x	1/2x	1/2x	1/2x	1/2x	2x	1/2x	1/2x	1/2x	1/2x	1/2x	1/2x
9	1/2xMS_2x$ZnSO_4$	1/2x	1/2x	1/2x	1/2x	1/2x	1/2x	1/2x	1/2x	2x	1/2x	1/2x	1/2x	1/2x	1/2x
10	1/2xMS_2xH_3BO_3	1/2x	1/2x	1/2x	1/2x	1/2x	1/2x	1/2x	1/2x	1/2x	2x	1/2x	1/2x	1/2x	1/2x
11	1/2xMS_2xKI	1/2x	1/2x	1/2x	1/2x	1/2x	1/2x	1/2x	1/2x	1/2x	1/2x	2x	1/2x	1/2x	1/2x
12	1/2xMS_2xNa_2MoO_4	1/2x	1/2x	1/2x	1/2x	1/2x	1/2x	1/2x	1/2x	1/2x	1/2x	1/2x	2x	1/2x	1/2x
13	1/2xMS_2x$CuSO_4$	1/2x	1/2x	1/2x	1/2x	1/2x	1/2x	1/2x	1/2x	1/2x	1/2x	1/2x	1/2x	2x	1/2x
14	1/2xMS_2x$CoCl_2$	1/2x	1/2x	1/2x	1/2x	1/2x	1/2x	1/2x	1/2x	1/2x	1/2x	1/2x	1/2x	1/2x	2x
15	1/2xMS_2xKNO_3_2xNH_4NO_3	2x	2x	1/2x	1/2x	1/2x	1/2x	1/2x	1/2x	1/2x	1/2x	1/2x	1/2x	1/2x	1/2x

Table S4. List of PCR Primers for Q-PCR

	Primer name	Sequence	Reference
For qPCR of *GTL1*	GTL1-627F2	ATGGAATTGTTTGAAGGTTTGG	Breuer et al 2009
	GTL1-627R2	GACATGACCTCGTGTTCTCG	Breuer et al 2009
For qPCR of *DF1*	DF1F5KN	GACATGGGAATAGCGTTTCG	Shibata et al 2018
	DF1R4KN	TCGGCATAACTGTCGTTACC	Shibata et al 2018
For qPCR of *RSL1*	RSL1_FWD	TCGTACCGCTACTCGGCTTCTT	Rymen et al 2017
	RSL1_REV	CAATAAACGGCCTTTCACGGGAGA	Rymen et al 2017
For qPCR of *RSL2*	RSL2_FWD	CTCGTCCCCAATGGAACAAAGGTC	Rymen et al 2017
	RSL2_REV	GCAATCGGCGCATACATCCATAGA	Rymen et al 2017
For qPCR of *RSL3*	RSL3_FWD	TCGTCCCTAATGGAACAAAGGTTG	Rymen et al 2017
	RSL3_REV	GGCCAATGTCCATTCCGTTGTAAG	Rymen et al 2017
For qPCR of *RSL4*	qRSL4-F	AGGCAAAACTAGAGCCACCA	Shibata et al 2018
	qRSL4-R	ATCGACTTTTGTCCCGTTTG	Shibata et al 2018
For qPCR of *RHD6*	qRHD6-F	TCACGAGAGCTTTCCTCCTC	Shibata et al 2018
	qRHD6-R	TGAAGCCGTAGCTCATGTTG	Shibata et al 2018
For qPCR of *LRL1*	qLRL1-F	TCTCAAATCTCCGAGGCTGG	This study
	qLRL1-R	TTTGGCCACTTGATGTTCCG	This study
For qPCR of *LRL2*	qLRL2-F	GATACCGGCGTTCCTTTGTC	This study
	qLRL2-R	AACGGAGGGAGCGTCTTTAA	This study
For qPCR of *LRL3*	qLRL3-F	AGATTGGGAGGTGCAGGATC	This study
	qLRL3-R	TCCATCAGTTTCGCCACTCT	This study
For qPCR of *EXPA7*	qEXPA7-F	TTAACAGCGGCTACGGACTG	This study
	qEXPA7-R	GGCAAAGATTGGTGGCTGTG	This study
For qPCR of *GL2*	qGL2-F	CCGATGATCTCCACCTCGAA	This study
	qGL2-R	GTGTTCTTGATCGTCGGAGC	This study
For qPCR of *UBQ10*	UBQ10-F	GAAGTGGAAAGCTCCGACAC	Shibata et al 2018
	UBQ10-R	TTAGAAACCACCACGAAGACG	Shibata et al 2018
For qPCR of *HEL*	HEL_hk_Fw	CCATTCTACTTTTTGGCGGCT	Rymen et al 2017
	HEL_hk_Rv	TCAATGGTAACTGATCCACTCTGATG	Rymen et al 2017

Table S5. List of PCR Primers for cloning

**Promoter cloning for LUC assay**		
RSL4 promoter	SacII-RSL4pro-1500-F	ATACCGCGGAGGTGCAATGTACCGTAACC
	BamHI-RSL4pro-R	ATAGGATCCCGCTCTAACTGATCAACTCTTG
	HindIII-RSL4p-1000-F	TCAGTAAGCTTGTGTTGAATTCGCCTTATTTGC
	HindIII-RSL4p-500-F	TCAGTAAGCTTTGATTCCACAAATCGTGCAT
	HindIII-RSL4p-250-F	TCAGTAAGCTTCATGCATGGCTTCGTTTCAC
	HindIII-RSL4p-150-F	TCAGTAAGCTTCATCACCAAATCTTCCTTGAG
	HindIII-pGEM-R	ATCATAAGCTTCCTATAGTGAGTCGTATTAC
RHD6 promoter	RHD6 proF	AAAGAATGGGCCGAATGTC
	RHD6 proR	TAGACACTAATAAGTTTGATAAGTGA
RSL4 promoter	SacII RSL4pro Fw	AAAGTAGCTAACCCGCGGGTGTGTGCATGCATGTGTGT
	BamHI RSL4pro RV	AAATTAAAGCTAACGGATCCCGCTCTAACTGATCAACTCTTGCC

**TF cloning forLUC assay**		
DF1	SmaI-DF1start-F2	ATATTCCCGGGATGATGCAACTGGGTGGTGG
	SmaI-DF1stop-R	ATTTACCCGGGTTACAGATTATTCGTCGTCTTG

**RSL4 cloning for overexpression lines**		
genomic *RSL4* and *RSL4* cDNA	attB1-RSL4-F	GGGGACAAGTTTGTACAAAAAAGCAGGCTGCATGGACGTTTTTGTTGATGGTG
	attB2-RSL4_noStop-R	GGGGACCACTTTGTACAAGAAAGCTGGGTCCATAAGCCGAGACAAAAGG
	attB2-RSL4_withStop-R	GGGGACCACTTTGTACAAGAAAGCTGGGTCTCACATAAGCCGAGACAAAAGG

**CoIP**		
EGFP-3xFLAG	attB1-EGFP-F	GGGGACAAGTTTGTACAAAAAAGCAGGCTTAatggtgagcaagggcgag
	attB2-3xFLAG-EGFP-R	GGGGACCACTTTGTACAAGAAAGCTGGGTTCTACTTGTCATCGTCATCCTTGTAATCGATGTCATGATCTTT ATAATCACCGTCATGGTCTTTGTAGTCgtacagctcgtccatgccgtg
RHD6-3xHA	attB1-RHD6cds-F	GGGGACAAGTTTGTACAAAAAAGCAGGCTTAATGGCACTCGTTAATGACCATCC
	attB2-3xHA-RHD6cds-R	GGGGACCACTTTGTACAAGAAAGCTGGGTTTTAagcgtaatctggaacgtcatatggataggatcctgcatagtccgggacgtcata gggatagcccgcatagtcaggaacatcgtatgggtaATTGGTGATCAGATTCGAATTCCTGTCT
GTL1-3xFLAG	attB1-GTL1-F	GGGGACAAGTTTGTACAAAAAAGCAGGCTTAATGGAGCAAGGAGGAGGTGG
	attB2-GTL1_TAIR_L-3xFLAG-R1	GTAATCGATGTCATGATCTTTATAATCACCGTCATGGTCTTTGTAGTCCTGAACCATTGTCAAGAAAGG
	attB2-GTL1_TAIR_L-3xFLAG-R2	GGGGACCACTTTGTACAAGAAAGCTGGGTTTTACTTGTCATCGTCATCCTTGTAATCGATGTCATGATCT

Reference

Breuer C, Kawamura A, Ichikawa T, Tominaga-Wada R, Wada T, Kondou Y, Muto S, Matsui M, Sugimoto K. **2009**. The Trihelix Transcription Factor GTL1 Regulates Ploidy-Dependent Cell Growth in the Arabidopsis Trichome. *The Plant Cell* **21**: 2307–2322.

Menand B, Yi K, Jouannic S, Hoffmann L, Ryan E, Linstead P, Schaefer DG, Dolan L. **2007**. An Ancient Mechanism Controls the Development of Cells with a Rooting Function in Land Plants. *Science* **316**: 1477–1480.

Rymen B, Kawamura A, Schäfer S, Breuer C, Iwase A, Shibata M, Ikeda M, Mitsuda N, Koncz C, Ohme-Takagi M, *et al.* **2017**. ABA Suppresses Root Hair Growth via the OBP4 Transcriptional Regulator. *Plant Physiology* **173**: 1750–1762.

Shibata M, Breuer C, Kawamura A, Clark NM, Rymen B, Braidwood L, Morohashi K, Busch W, Benfey PN, Sozzani R, *et al.* **2018**. GTL1 and DF1 regulate root hair growth through transcriptional repression of ROOT HAIR DEFECTIVE 6-LIKE 4 in Arabidopsis. *Development* **145**: dev159707.

Yi K, Menand B, Bell E, Dolan L. **2010**. A basic helix-loop-helix transcription factor controls cell growth and size in root hairs. *Nature Genetics* **42**: 264–267.
